# A ‘Plug and Play’ Platform for the Production of Diverse Monoterpene Hydrocarbon Scaffolds in *Escherichia coli*

**DOI:** 10.1002/slct.201600563

**Published:** 2016-06-21

**Authors:** Nicole G. H. Leferink, Adrian J. Jervis, Ziga Zebec, Helen S. Toogood, Sam Hay, Eriko Takano, Nigel S. Scrutton

**Affiliations:** [a] SYNBIOCHEM, Manchester Institute of Biotechnology, Faculty of Life Sciences, University of Manchester, Manchester M1 7DN (UK)

**Keywords:** Chemical Diversity, Monoterpene Cyclases/Synthases, Production Platform, Synthetic Biology, Terpenoids

## Abstract

The terpenoids constitute one of the largest and most diverse classes of natural compounds with applications as pharmaceuticals, flavorings and fragrances, pesticides and biofuels. Synthetic biology is ideally placed to create new routes to this chemical diversity and facilitation of new compound discovery. The C10 monoterpenoids display a huge structural diversity produced from a single substrate, geranyl diphosphate, by a family of monoterpene cyclases and synthases (mTC/S). Here we employ a library of mTC/S in a single ‘plug and play’ platform system for the production of over 30 different monoterpenoids in Escherichia coli by fermentation on glucose. These products include several compounds never before produced in engineered microbes demonstrating the power of this approach to rapidly create routes to structural diversity.

Terpenoids are one of the largest and most diverse classes of natural products, with over 55,000 described structures.[1] Most of these are produced by plant species where they are the major component of essential oils primarily produced in response to abiotic and biotic stress.[2] The global market value of essential oils (> $5 billion), and the terpene/terpenoids therein, is a result of their diverse use as pharmaceuticals, flavorings, fragrances, antimicrobials, pesticides and household products as well as their potential as new biofuels.[3] All terpenoids are naturally synthesized from the universal C5 isoprenoid precursors isopentanyl diphosphate (IPP) and dimethylallyl diphosphate (DMAPP), the products of either the methylerythritol 4-phosphate (MEP) pathway, or the mevalonate-dependent (MVA) pathway (Figure 1 A). Condensation of IPP and DMAPP by a prenyltransferase followed by the activity of a single terpene synthase is sufficient for the production of many terpenoids. Studies over the last decade have focused on the optimization of these pathways in microbial chassis (predominantly *E. coli* and *Saccharomyces cerevisiae*) employing a synthetic biology approach for the tractable production of terpenoids from cheap feedstocks, via fermentation.[4] Not only does synthetic biology offer a green technology solution for production and ease of access to scarce compounds; it also has the capability to create further diversity in new-to-nature derivatives. Indeed a recent study was successful in rapidly producing a combinatorial genetic platform for the production of a diverse diterpene library containing novel compounds.[5]

Many attractive target terpenoids are members of the C10 monoterpenoids and a number have been produced by microbial chassis including limonene,[6] pinene,[7] geraniol[8] and menthol.[9] The monoterpenoids offer a relatively simple route to diverse linear, monocyclic and bicyclic structures, attractive hydrocarbon skeletons with the potential for further derivatization (Figure 1 A).

The monoterpenoids are synthesized through the activity of the large monoterpene cyclase/synthase (mTC/S) enzyme family. Members of this family employ the class I terpene cyclase fold[10] and catalyze ‘high’ energy cyclisation reactions involving unstable carbocation intermediates originating from a single precursor substrate. All mTC/S employ the same reaction mechanism, which is initiated by the metal dependent ionization of the linear C10 isoprenoid precursor geranyldiphosphate (GPP). The resulting carbocation then reacts along one of several channels to form linear, monocyclic and bicyclic structures (Figure 1 A). This remarkable chemical diversity is achieved because a single carbocation can undergo a range of cyclizations and isomerization before the reaction terminates by either deprotonation or water capture.[11] Chemical diversity is not only the result of the large number of mTC/S that exist, but also from the ability of a single mTC/S to create multiple products.[11] After the initial carbocation formation, the enzyme itself does little else than provide a productive template for the cyclization cascade and stabilize the reactive carbocation intermediates.[12] This is reflected by the facts that little correlation can be observed between the active site sequence and cyclization type, and closely related enzymes with varying product profiles.[10, 12]

Here we present the construction of a flexible monoterpenoid production platform in *E. coli* employing a library of mTC/S implemented for the production of over 16 linear, monocyclic and bicyclic monoterpenoid hydrocarbon scaffolds, labelled throughout (**1**-**16**).

To create a diverse array of monoterpenoids, 37 mTC/Ss from a range of plant species (Table S3 in the Supporting Information) were selected for incorporation in the platform. Selection was based on the published product profile, source, and previous successful recombinant expression in *E. coli.* We aimed at sufficient enzyme diversity to cover the formation of the hydrocarbon scaffolds shown in Figure 1 and typically two mTC/S were selected for each target compound including enantiomers which increased the total number of targets to 19. The resulting mTC/S library has a large sequence diversity and the potential to create diverse monoterpenoid production profiles.

Multiple *E. coli* strains (including K-12, B and W strains) were screened for limonene production when carrying a previously optimised limonene production pathway (plasmid pJBEI-6410) consisting of an inducible hybrid MVA pathway and genes encoding an isopentenyl diphosphate isomerase (*idi*), a truncated GPP synthase (*GPPS*) and a truncated limonene synthase (*lim-S*)[6a] The highest titres were produced by the K-12 strain DH5α, which was selected as our production chassis (Figure S2 in the Supporting Information).

To create a flexible system for switching mTC/S two gene modules were constructed (Figure 1B). The first module (plasmid pMVA) consists of the MVA pathway and *idi* gene under regulation of IPTG-inducible promoters from pJBEI-6410[6a] and the second (plasmid series pGPPSmTC/S, See Table S4 in the Supporting Information) consists of a refactored, N-terminal truncated *Abies grandis* GPPS gene (Agtr*GPPS2*) and mTC/S genes under regulation of a tetracycline-inducible promoter. Design of the pGPPSmTC/S series was such that the mTC/S genes with cognate ribosome binding sites (RBS) were flanked by unique restriction sites allowing a ‘plug-and-play’ switching of mTC/S genes. All mTC/S genes with RBS were introduced after PCR amplification from a pET-based expression vector using a single set of PCR primers creating a set of 37 different plasmids (See Supporting Information). Co-transformation of pMVA and the pGPPSmTC/S series resulted in 37 different *E. coli* monoterpenoid production strains, together constituting the monoterpenoid production platform. Strains were grown in a two-phase shake flask system using glucose as the feedstock and *n*-nonane as the organic phase. Products, accumulating in the organic phase (no monoterpenoids were detected in the culture medium after extraction with ethyl acetate - data not shown), were identified and quantified by GC-MS analysis by comparison to retention times and MS fragmentation patterns displayed by authentic standards wherever possible (Figure S3 in the Supporting Information). In the absence of authentic standards, MS fragmentation patterns were entered into the NIST mass spectral library for identification of a potential match. GC-MS profiles of the organic overlays obtained for each strain are shown in the Supporting Information (Figure S4). Initial tests of the system used the (*S*)-limonene synthase from *Mentha spicata* (SlimS_Ms) successfully displayed titres comparable to strains containing plasmid pJBEI-6410 (~ 500 mg L^−1^; Figure S2 in the Supporting Information).

Across the 37 strains we were able to detect a total of 31 different monoterpenoids, 11 linear, 7 monocyclic and 13 bicyclic compounds (Tables S6-S8 in the Supporting Information). Across the 37 strains 16 of the initial target monoterpenoids were produced by at least one mTC/S with a total of 20 mTC/S producing detectable quantities of their predicted primary product(s) (Figure 2). Crucially we were able to generate several novel scaffolds which have not previously been produced in engineered microbes. These include γ-terpinene (**6**; 197 mg L_org_^−1^), fenchol (**15**; 69 mg L_org_^−1^), α-terpineol (**9**; 38 mg L_org_^−1^), sabinene (**12**; 77 mg L_org_^−1^), (*E*)-β-ocimene (**2**; 57 mg L_org_^−1^), camphene (**14**; 12 mg L_org_^−1^), β-phellandrene (**7**; 7.4 mg L_org_^−1^), and terpinolene (**5**; 3.4 mg L_org_^−1^). In addition to these target compounds, several other new scaffolds were detected in small amounts as by-products (Figure S4 and Tables S5-S9 in the Supporting Information).

Furthermore, using our platform we were able to exceed production levels for most monoterpenoid scaffolds previously produced in *E. coli* and/or yeast (Figure S5 in the Supporting Information). The highest producing strains containing the mTC/S (-)aPinS_Pt and SLimS_Ms respectively, resulted (-)-α-pinene (**10**) and *S-*limonene (**8**) titres of ~ 550-600 mg L_org_^−1^ with 81 % and 96 % purity, respectively. These levels exceed previously published values for pinene (32.0 mg L_org_^−1^),[7] and limonene (435 mg L_org_^−1^)[6a] produced under similar two-phase shake-flask culture conditions. These titres provide an excellent basis for scale-up using fermentation conditions, as previous fed-batch fermentation experiments with engineered *E. coli* resulted in limonene and α-pinene titres of 1.35 and 0.97 g L^−1^ respectively.[13]

At the other end of the spectrum our platform produced small but significant amounts of linalool (**3**; 0.8 mg/L_org_), which is in the same range of the value of 0.1 mg L^−1^ reported for previous exogenous linalool production in *S. cerevisiae.*[14]

Most synthases yielded a mixture of products, with the main product constituting between 30–99 % of the total product mixture (Figure 2B). In addition to the expected monoterpenoid products based on the mTC/S used, geraniol (**1**) and its derivatives nerol, neral, geranial, citronellol, and citronellal, the so-called geranoids, were detected in the *n*-nonane overlays of most cultures tested, with the titres depending on the amount of target monoterpenoids produced. A high monoterpenoid titre (> 100 mg L_org_^−1^) resulted in a low level of geranoid production (10-50 mg L_org_^−1^), whereas a low monoterpenoid titre could result in geranoid titres of > 140 mg L_org_^−1^ in the absence of a geraniol synthase. In the presence of a geraniol synthase (GerS_Pc), this figure increased to 350 mg L_org_^−1^, of which ~ 50 % is geraniol (**1**). (Figure S6 in the Supporting Information). A control strain containing the MVA pathway and GPPS, but no mTC/S did also produce geranoids (4.7 ± 2.6 mg L_org_^−1^), albeit at a much lower level than seen in some of the production strains. It has been demonstrated that an endogenous *E. coli* alkaline phosphatase (PhoA) can convert GPP into geraniol in the presence of a heterologous MVA pathway and GPPS,[15] which can subsequently be converted into other geranoids by endogenous dehydrogenation and isomerization.[8] However, the production strain used in this study, *E. coli α*-Select, is PhoA negative indicating other endogenous pathways are also capable of converting GPP to geraniol and beyond. The higher geraniol titres from some production strains (e. g. TerS_Pm and OciS_Am) implies the expressed mTC/S are capable of geraniol production. The unidentified endogenous pathway is also likely responsible for the conversion of farnesyl diphosphate (FPP), which is natively produced by *E. coli*,[16] into farnesol,[17] another terpene by-product detected in significant amounts (10-40 mg L_org_^−1^) in almost all strains used in this study (Figure S6 in the Supporting Information). However, it has been demonstrated that endogenous FPP synthesis does not affect monoterpenoid yield in *E. coli* despite competing for IPP and DMAPP.[13a]

In summary, using our scalable ‘plug-and-play’ *E. coli* monoterpenoid production platform we were able to produce over 30 different linear, monocyclic, and bicyclic monoterpenoid scaffolds from glucose using a heterologous MVA pathway and interchangeable mTC/S. This platform provides an excellent basis for further optimisation and diversification into other valuable monoterpenoids. Future efforts will be directed towards further balancing of the MVA pathway and eliminating the formation of unwanted by-products resulting in robust monoterpenoid production strains that can be exploited for the stereoselective biosynthesis of this important group of compounds.

## Experimental Section

Generation of the various constructs, monoterpenoid production conditions, and product analysis are described in the Supporting Information.

## Supplementary Material

Supporting information for this article is available on the WWW under http://dx.doi.org/10.1002/slct.201600563

SI

## Figures and Tables

**Figure 1:**
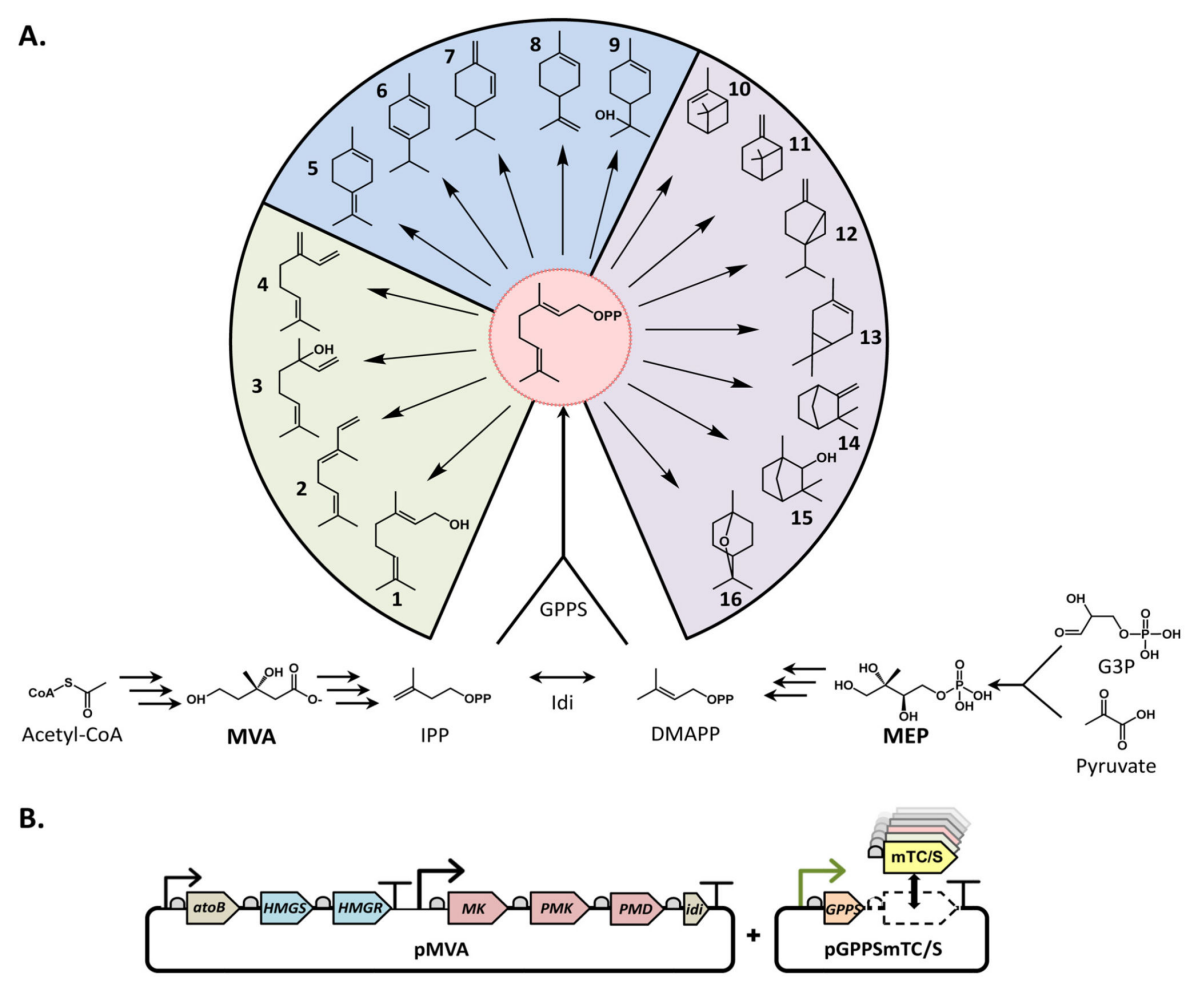
Platform for diverse monoterpenoid production in *E. coli*. **A.** Exogenous, hybrid MVA pathway and endogenous MEP pathway lead to the production of GPP. GPP is the sole substrate for all monoterpenoid synthases/cyclases leading to a diverse hydrocarbon skeleton; **1** geraniol, **2** (*E*)-β-ocimene, **3** linalool, **4** β-myrcene, **5** terpinolene, **6** γ-terpinene, **7** β-phellandrene, **8** limonene, **9** α-terpineol, **10** α-pinene, **11** β-pinene, **12** sabinene, **13** 3-carene, **14** camphene, **15** fenchol, and **16** 1,8-cineole. **B.** Plasmid organisation for the production of monoterpenoids in *E. coli.* pMVA leads to IPP and DMAPP formation, plasmid pGPPSmTC/S leads to GPP and monoterpenoid formation. Multiple mTC/S can be exchanged at position 2 to produce analogous production strains.

**Figure 2:**
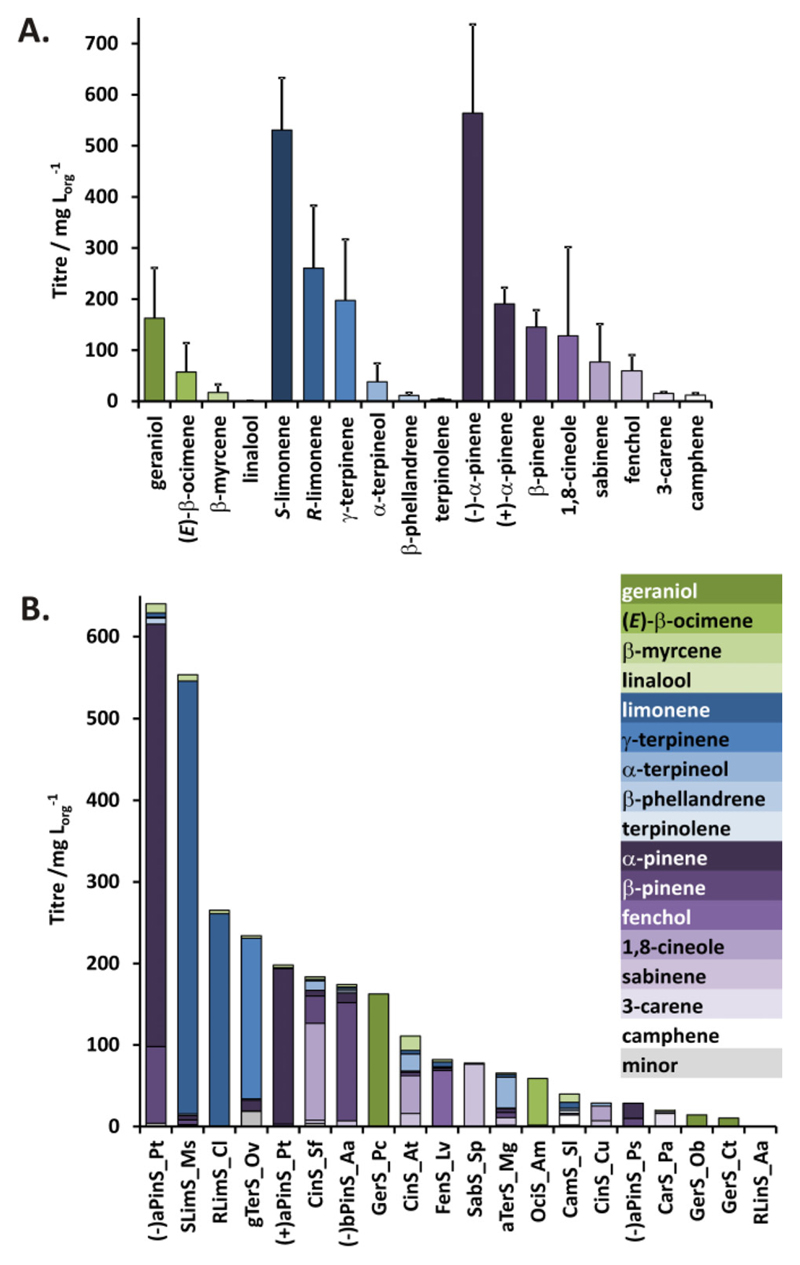
Monoterpenoid titres achieved in this study using the *E. coli* platform. **A.** Highest titres observed for each monoterpenoid hydrocarbon scaffold. Error bars represent the standard deviation of at least 3 biological replicates. **B.** Product profiles observed for all active mTC/S tested in the platform. Linear monoterpenoids are in shades of green, monocyclic monoterpenoids in blue, and bicyclic monoterpenoids in purple. See full enzyme names and break-down of the product profiles in the Supporting Information.
