# Supplementary material for: A ‘Plug and Play’ Platform for the Production of Diverse Monoterpene Hydrocarbon Scaffolds in Escherichia coli
Source: ChemistrySelect. Author manuscript; Available in PMC 2018 May 11. (PMC5947754; doi:10.1002/slct.201600563)
Supplement: SI [file NIHMS69571-supplement-SI.pdf]

Chemistry SELECT

Supporting Information

© Copyright Wiley-VCH Verlag GmbH & Co. KGaA, 69451 Weinheim, 2016

A 'Plug and Play' Platform for the Production of Diverse Monoterpene Hydrocarbon Scaffolds in *Escherichia coli.*

Nicole G. H. Leferink, Adrian J. Jervis, Ziga Zebec, Helen S. Toogood, Sam Hay, Eriko Takano, and Nigel S. Scrutton*

Supporting Information

Table of contents

Experimental Section

Bacterial strains and media 2
Construction of plasmids 2
Monoterpene production conditions, product capture and detection 3
Figure S1: Plasmid maps of pMVA and pBbB2a-trAgGPPS(co)-trMsLS 4
Table S1: Strains used in this study 5
Table S2: Primers used in this study 6
Table S3: Monoterpene synthases used in this study 7
Table S4: Plasmids used in this study 8

Supporting Results

Figure S2**:** Limonene production titres in different *E. coli* strains 9
Figure S3: GCMS analysis of authentic monoterpenoid standards used in this study 10
Figure S4: GCMS analysis of monoterpenoid production strains 12
A) (-)aPinS_Pt 13
B) SLimS_Ms 14
C) RLimS_Cl 15
D) gTerS_Ov 16
E) (+)aPinS_Pt 17
F) CinS_Sf 18
G) (-)bPinS_Aa 19
H) GerS_Pc 20
I) CinS_At 21
J) FenS_Lv 22
K) SabS_Sp 23
L) aTerS_Mg 24
M) OciS_Am 25
N) CamS_Sl 26
O) CinS_Cu 27
P) (-)aPinS_Ps 28
Q) CarS_Pa 29
R) GerS_Ob 30
S) GerS_Ct 31
T) RLinS_Aa 32
Table S5: Product profiles and monoterpenoid titres of production strains 33
Table S6: Overview of all linear monoterpenoids produced 34
Table S7: Overview of all monocyclic monoterpenoids produced 35
Table S8: Overview of all bicyclic monoterpenoids produced 36
Table S9: Overview of all additional terpenoids produced 37
Figure S5: Titres produced in the platform vs. previously published titres 38
Figure S6: Monoterpenoid, geranoid and farnesol titres for each strain 39

References 40

Experimental Section

Bacterial strains and media

All *E. coli* strains (Table S1) were routinely grown in Lysogeny Broth (LB) or on LB agar plates including antibiotic supplements as appropriate (ampicillin, 100 µg ml^{-1}; kanamycin, 50 µg mL^{-1}). Cloning and plasmid propagation was performed using *E. coli* α-Select (Bioline).

Construction of plasmids

To construct plasmid pMVA (Figure S1, panel A), the hybrid MVA pathway and *E. coli idi* (*Ecidi*), including 5' PlacUV5 was PCR amplified using primers MVA-F and MVA-R (Table S9) from pJBEI-6410[1]. The p15a origin of replication was similarly amplified using primers p15a-F and p15a-R and a kanamycin resistance cassette was amplified from pBbA1k-RFP[2] using primers kan-F and kan-R. These fragments were phosphorylated and assembled using the ligase cycling reaction with bridging oligos brMBAori, brorikan, crkanMVA (Table S2) as previously described[3].

pBbB2a-trAgGPPS(co)-trMsLS (Figure S1, panel B) was constructed by insertion of *Abies grandis GPPS*2 (GPPS_Ag) and *Mentha spicata* (*limS*_Ms) into plasmid pBbB2a-RFP[2]. The predicted plastidial signal sequence was removed from both LimS_Ms and GPPS_Ag, as previously reported[1, 4] and were codon-optimised for expression in *E. coli* (GeneArt, Life Technologies) and The Ribosome Binding Site Calculator[5] was used to design bespoke 5' untranslated regions (UTRs) encoding a RBS sites for each gene (A.U.=15,000) and to remove any potential aberrant, mid-gene translational start sites (A.U.<1,000). The limS_Ms unit (including 5' UTR) was flanked with HindIII and BamHI restriction sites and both gene units were synthesised together (GeneArt) flanked by EcoRI and XhoI restriction sites, for subcloning into pBbB2a-GFP.

For production of other monoterpenoids the limS_Ms gene was replaced by genes encoding various other monoterpene synthases (Table S3). Synthetic genes encoding monoterpene synthases without plastidial signal sequences were codon-optimised for expression in *E. coli* and sub-cloned into the NcoI-XhoI restriction sites of pETM-11 (a modified version of the pET24-b vector, Novagen), fused to a TEV protease cleavable N-terminal His_6-tag. Monoterpene synthase genes, including RBS, were amplified from the pETM-11 plasmids using primers pETM-11_Fw and pETM-11_Rv (Table S2). PCR fragments were cloned between the HindIII and BamHI sites of the pBbB2a-trAgGPPS(co)-trMsLS, thereby removing the original limS_Ms gene. The 3 geraniol synthase genes (GerS_Ob, GerS_PC and GerS_Ct) were cloned by InFusion (Takara) after PCR amplification (primer pairs trObGES_Fv-over / trObGES_Rv-over, trPcGES_Fv-over / trPcGES_Rv-over, trCtGES_Fv-over / trCtGES_Rv-over, Table S2) into plasmid pGPPSmTC/S15 which was PCR linearized (pLimS_inverse_rev/ pLimS_inverse_Fw, Table S2).

The control plasmid pBbGPPS, without mTC/S was constructed by amplification of the complete tp pSBC000339 plasmid except LimS_Ms using primers pBbGPPS_Fw and pBbGPPS_Rv, the resulting PCR product was digested with HindIII and subsequently self-ligated.

Monoterpene production conditions, product capture and detection

Expression strains were inoculated with freshly transformed colonies into 3 ml terrific broth (TB) supplemented with 0.4 % glucose and antibiotics in 28 ml glass screw capped vials. Cultures were grown for 7 h at 37 °C with shaking at 200 rpm before transferring to 30 °C and induction with 50 µM (isopropyl β-D-1-thiogalactopyranoside) IPTG and 25 nM anhydro-tetracycline (aTet) and overlaid with a 20 % *n*-nonane layer followed by incubation for 72 h with shaking at 200 rpm.

A_{600} readings were taken of the aqueous phase, and nonane layers were harvested and clarified by centrifugation (14,000 rpm, 3 min, 4°C), dried over anhydrous $MgSO_4$ and mixed 1:1 with ethyl acetate containing 0.1 % sec-butyl benzene.

Monoterpene products were analysed by GCMS using an Agilent Technologies 7890B GC equipped with an Agilent Technologies 5977A MSD. The products were separated on a DB-WAX column (30 m x 0.32 mm i.d., 0.25 µM film thickness, Agilent Technologies). The injector temperature was set at 240°C with a split ratio of 20:1 (1 µL injection). The carrier gas was helium with a flow rate of 1 mL/min and a pressure of 5.1 psi. The following oven program was used: 50°C (1 min hold), ramp to 68°C at 5°C/min (2 min hold), and ramp to 230°C at 25°C/min (2 min hold). The ion source temperature of the mass spectrometer (MS) was set to 230°C and spectra were recorded from m/z 50 to m/z 250. Compound identification was carried out using authentic standards and comparison to reference spectra in the NIST library of MS spectra and fragmentation patterns.

Monoterpenoids were quantified using authentic standards wherever possible, using experimentally determined relative response factors in relation to the internal standard used. In the absence of an authentic standard concentrations were estimated using a relative response factor of 1.

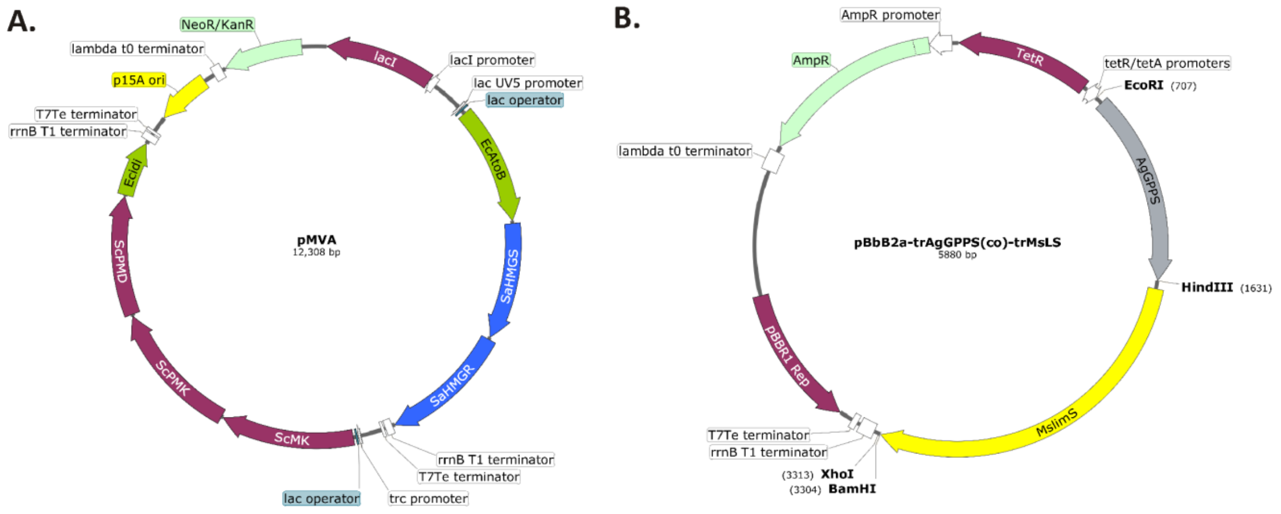

Figure S1: Plasmid maps of pMVA (A) and pBbB2a-trAgGPPS(co)-trMsLS (B). Figures were prepared using the SnapGene software package.

Table S1: Strains used in this study

Strain name	Strain	Reference
**Chassis**		
DH5α (α-Select)	K-12	Bioline
MG1655	K-12	[6]
W3110	K-12	[7]
DH1	K-12	[8]
MDS42 Meta	K-12	Scarab Genomics
BL21 (λDE3)	B	New England Biolabs
Mach1	W	Thermo-Fisher
**Production strain**		
DH5α (α-Select)	K-12	Bioline

Table S2: Primers used in this study

Primer	Sequence (5'-3')
JBEIMVA-F	ATTTAGAAAAATAAACAAATAGGGGTTC
JBEIMVA-R	TTATTTAAGCTGGGTAAATGCAG
p15a-F	GGATCCAAACTCGAGTAAGG
p15a-R	GGTAACTGTCAGACCAAGTTTAC
kan-F	TCGACGTCGGAATTGCCAGC
kan-R	TCAGAAGAACTCGTCAAGAAGG
brMVAori	GCCAGAAAACGATTATCTGCATTTACCCAGCTTAAATAAGGATCCAAACTCGAGTAAGGATCTCCAG
brorikan	AAGCGAGCTCGTAAACTTGGTCTGACAGTTACCTCAGAAGAACTCGTCAAGAAGGCGATAGAAGGC
brkanMVA	CCCCAGCTGGCAATTCCGACGTCGAATTTAGAAAAATAAACAAATAGGGGTTCCGCGCACATTTC
pETM-11_Fw	CGCGCGAAGCTTAATAATTTTGATTTAAC
pETM-11_Rv	ATGGATCCCTTTGTTAGCAGCCGGATC
pBbGPPS_Fw	CCCAAGCTTAAACTCGAGTAAGGATCTCC
pBbGPPS_Rv	CCCAAGCTTAATTCTGACGAAATGC
pLimS_inverse_rev	GGCGCCCTGAAAATAAAGAT
pLimS_ inverse_Fw	TAATAACTCGAGCACCACCACC
trObGES_Rv	CTGGGTAAAAAACAGGGCAT
trObGES_Fv	ATGGAAGAAAGCAGCAGCAA
trObGES_Fv-over	ATCTTTATTTTCAGGGCGCCATGGAAGAAAGCAGCAGCAA
trObGES_Rv-over	TGGTGGTGCTCGAGTTATTACTGGGTAAAAAACAGGGCAT
trPcGES_Fv-over	ATCTTTATTTTCAGGGCGCCATGGCACGTCGTAGCGGTAATTATCA
trPcGES_Rv-over	TGGTGGTGCTCGAGTTATTAAACATACGGCTCAAACATCAGA
trCtGES_Fv-over	ATCTTTATTTTCAGGGCGCCATGGCACGTCGTAGCGGTAACTATAAA
trCtGES_Rv-over	TGGTGGTGCTCGAGTTATTATGCGCTACCACCATCAACA

Table S3: Monoterpene synthase genes used in this study. All synthetic genes were codon optimised for use in *E. coli,* fused to a cleavable N-terminal His_6-tag, and, if present, the N-terminal plastid sequence was omitted.

**Name**	**Source**	**Main product**	**Uniprot nr.**	**Reference**
CamS_Ag	*Abies grandis*	Camphene	Q948Z0	[9]
CamS_Sl	*Solanum lycopersicum*	Camphene	G1JUH1	[10]
CarS_Pa	*Picea abies*	3-Carene	Q84SM8	[11]
CarS_Ps	*Picea sitchensis*	3-Carene	F1CKI6	[12]
CinS_Sf	*Salvia fruticosa*	1,8-Cineole	A6XH05	[13]
CinS_Cu	*Citrus unshiu*	1,8-Cineole	Q5CD82	[14]
CinS_At	*Arabidopsis thaliana*	1,8-Cineole	P0DI76	[15]
FenS_Ob	*Ocimum basilicum*	Fenchol	Q5SBP2	[16]
FenS_Lv	*Lavandula viridis*	Fenchol	T1RR72	This study
GerS_Pc	*Perilla citriodora*	Geraniol	Q4JHG3	[17]
GerS_Ob	*Ocimum basilicum*	Geraniol	Q6USK1	[18]
GerS_Ct	*Cinnamomum tenuipilum*	Geraniol	Q8GUE4	[19]
RLimS_Cl	*Citrus lemon*	(+)-(4*R*)-Limonene	Q8L5K3	[20]
RLimS_La	*Lavandula angustivolia*	(+)-(4*R*)-Limonene	Q2XSC6	[21]
SLimS_Ms	*Mentha spicata*	(-)-(4*S*)-Limonene	Q40322	[22]
RLinS_Aa	*Artemisia annua*	(-)-(3*R*)-linalool	Q9SPN0	[23]
SLinS_At	*Arabidopsis thaliana*	(+)-(3*S*)-linalool	Q84UV0	[24]
MyrS_Ag	*Abies grandis*	β-Myrcene	O24474	[9, 25]
MyrS_Ca	*Coffea arabica*	β-Myrcene	R4YXW8	[26]
MyrS_At	*Arabidopsis thaliana*	β-Myrcene	Q9ZUH4	[27]
OciS_Cu	*Citrus unshiu*	(*E*)-β-ocimene	Q5CD81	[14]
OciS_Am	*Antirrhinum majus*	(*E*)-β-ocimene	Q84NC8	[28]
PheS_Ag	*Abies grandis*	β-phellandrene	Q9M7D1	[9]
PheS_La	*Lavandula angustivolia*	β-phellandrene	E9N3U9	[29]
PheS_Sl	*Solanum lycopersicum*	β-phellandrene	C1K5M3	[30]
(-)aPinS_Ps	*Picea sitchensis*	(-)-α-pinene	Q84KL6	[31]
(-)aPinS_Pt	*Pinus taeda*	(-)-α-pinene	Q84KL6	[32]
(+)aPinS_Pt	*Pinus taeda*	(+)-α-pinene	Q84KL3	[32]
(-)bPinS_Aa	*Artemisia annua*	(-)-β-pinene	Q94G53	[33]
SabS_Ps	*Picea sitchensis*	Sabinene	F1CKJ1	[12]
SabS_Sp	*Salvia pomifera*	Sabinene	A6XH06	[13]
gTerS_Cs	*Coriandrum sativum*	γ-Terpinene	A0A059SVE6	[34]
gTerS_Ov	*Origanum vulgare*	γ-Terpinene	E2E2P0	[35]
aTerS_Mg	*Magnolia grandiflora*	α-Terpineol	B3TPQ7	[36]
aTerS_Sa	*Santalum album*	α-Terpineol	B5A434	[37]
TerS_Ob	*Ocimum basilicum*	Terpinolene	Q5SBP0	[16]
TerS_Pm	*Pseudotsuga menziesii*	Terpinolene	Q4QSN6	[38]

Table S4: Plasmids used in this study

Plasmid reference	Plasmid name	Description (Origin of replication, Antibiotic marker, Reference(s), Promoters and Operons)	Reference(s)
pJBEI-6410	pBbA5a-MTSAe-T1f-MBI(f)-T1002i-Ptrc-trGPPS(co)-LS	p15A, Ampr, PlacUV5, MTSA, T1, MBI-f, T1002, Ptrc, trGPPS, LS	[1]
pSBC000338	pBbA5a-MTSAe-T1f-MBI(f)-T1002i	p15A, Kanr, PlacUV5, MTSA, T1, MBI-f, T1002	This study
pSBC000339	pBbB2a-trAgGPPS(co)-trMsLS	pBBR, Ampr, Ptet, trAgGPPS(co)-trMsLS	This study
pGPPS	pBbB2a-trAgGPPS(co)	pBBR, Ampr, Ptet, trAgGPPS(co)	This study
pGPPSmTC/S1	pBbB2a-trAgGPPS(co)-trCamS_Ag	pBBR, Ampr, Ptet, trAgGPPS(co)- trCamS_Ag	This study
pGPPSmTC/S2	pBbB2a-trAgGPPS(co)-trCamS_Sl	pBBR, Ampr, Ptet, trAgGPPS(co)- trCamS_Sl	This study
pGPPSmTC/S3	pBbB2a-trAgGPPS(co)-trCarS_Pa	pBBR, Ampr, Ptet, trAgGPPS(co)- trCarS_Pa	This study
pGPPSmTC/S4	pBbB2a-trAgGPPS(co)-trCarS_Ps	pBBR, Ampr, Ptet, trAgGPPS(co)- trCarS_Ps	This study
pGPPSmTC/S5	pBbB2a-trAgGPPS(co)-trCinS_Sf	pBBR, Ampr, Ptet, trAgGPPS(co)- trCinS_Sf	This study
pGPPSmTC/S6	pBbB2a-trAgGPPS(co)-trCinS_Cu	pBBR, Ampr, Ptet, trAgGPPS(co)- trCinS_Cu	This study
pGPPSmTC/S7	pBbB2a-trAgGPPS(co)-trCinS_At	pBBR, Ampr, Ptet, trAgGPPS(co)- trCinS_At	This study
pGPPSmTC/S8	pBbB2a-trAgGPPS(co)-trFenS_Ob	pBBR, Ampr, Ptet, trAgGPPS(co)- trFenS_Ob	This study
pGPPSmTC/S9	pBbB2a-trAgGPPS(co)-trFenS_Lv	pBBR, Ampr, Ptet, trAgGPPS(co)- trFenS_Lv	This study
pGPPSmTC/S10	pBbB2a-trAgGPPS(co)-trGerS_Pc	pBBR, Ampr, Ptet, trAgGPPS(co)- trGerS_Pc	This study
pGPPSmTC/S11	pBbB2a-trAgGPPS(co)-trGerS_Ob	pBBR, Ampr, Ptet, trAgGPPS(co)- trGerS_Ob	This study
pGPPSmTC/S12	pBbB2a-trAgGPPS(co)-trGerS_Ct	pBBR, Ampr, Ptet, trAgGPPS(co)- trGerS_Ct	This study
pGPPSmTC/S13	pBbB2a-trAgGPPS(co)-trRLimS_Cl	pBBR, Ampr, Ptet, trAgGPPS(co)- trRLimS_Cl	This study
pGPPSmTC/S14	pBbB2a-trAgGPPS(co)-trRLimS_La	pBBR, Ampr, Ptet, trAgGPPS(co)- trRLimS_La	This study
pGPPSmTC/S15	pBbB2a-trAgGPPS(co)-trSLimS_Ms	pBBR, Ampr, Ptet, trAgGPPS(co)- trSLimS_Ms	This study
pGPPSmTC/S16	pBbB2a-trAgGPPS(co)-trRLinS_Aa	pBBR, Ampr, Ptet, trAgGPPS(co)- trRLinS_Aa	This study
pGPPSmTC/S17	pBbB2a-trAgGPPS(co)-trSLinS_At	pBBR, Ampr, Ptet, trAgGPPS(co)- trSLinS_At	This study
pGPPSmTC/S18	pBbB2a-trAgGPPS(co)-trMyrS_Ag	pBBR, Ampr, Ptet, trAgGPPS(co)- trMyrS_Ag	This study
pGPPSmTC/S19	pBbB2a-trAgGPPS(co)-trMyrS_Ca	pBBR, Ampr, Ptet, trAgGPPS(co)- trMyrS_Ca	This study
pGPPSmTC/S20	pBbB2a-trAgGPPS(co)-trMyrS_At	pBBR, Ampr, Ptet, trAgGPPS(co)- trMyrS_At	This study
pGPPSmTC/S21	pBbB2a-trAgGPPS(co)-trOciS_Cu	pBBR, Ampr, Ptet, trAgGPPS(co)- trOciS_Cu	This study
pGPPSmTC/S22	pBbB2a-trAgGPPS(co)- trOciS_Am	pBBR, Ampr, Ptet, trAgGPPS(co)- trOciS_Am	This study
pGPPSmTC/S23	pBbB2a-trAgGPPS(co)- trPheS_Ag	pBBR, Ampr, Ptet, trAgGPPS(co)- trPheS_Ag	This study
pGPPSmTC/S24	pBbB2a-trAgGPPS(co)- trPheS_La	pBBR, Ampr, Ptet, trAgGPPS(co)- trPheS_La	This study
pGPPSmTC/S25	pBbB2a-trAgGPPS(co)- trPheS_Sl	pBBR, Ampr, Ptet, trAgGPPS(co)- trPheS_Sl	This study
pGPPSmTC/S26	pBbB2a-trAgGPPS(co)- tr(-)aPinS_Ps	pBBR, Ampr, Ptet, trAgGPPS(co)- tr(-)aPinS_Ps	This study
pGPPSmTC/S27	pBbB2a-trAgGPPS(co)- tr(-)aPinS_Pt	pBBR, Ampr, Ptet, trAgGPPS(co)- tr(-)aPinS_Pt	This study
pGPPSmTC/S28	pBbB2a-trAgGPPS(co)- tr(+)aPinS_Pt	pBBR, Ampr, Ptet, trAgGPPS(co)- tr(+)aPinS_Pt	This study
pGPPSmTC/S29	pBbB2a-trAgGPPS(co)- tr(-)bPinS_Aa	pBBR, Ampr, Ptet, trAgGPPS(co)- tr(-)bPinS_Aa	This study
pGPPSmTC/S30	pBbB2a-trAgGPPS(co)- trSabS_Ps	pBBR, Ampr, Ptet, trAgGPPS(co)- trSabS_Ps	This study
pGPPSmTC/S31	pBbB2a-trAgGPPS(co)- trSabS_Sp	pBBR, Ampr, Ptet, trAgGPPS(co)- trSabS_Sp	This study
pGPPSmTC/S32	pBbB2a-trAgGPPS(co)- trgTerS_Cs	pBBR, Ampr, Ptet, trAgGPPS(co)- trgTerS_Cs	This study
pGPPSmTC/S33	pBbB2a-trAgGPPS(co)- trgTerS_Ov	pBBR, Ampr, Ptet, trAgGPPS(co)- trgTerS_Ov	This study
pGPPSmTC/S34	pBbB2a-trAgGPPS(co)- traTerS_Mg	pBBR, Ampr, Ptet, trAgGPPS(co)- traTerS_Mg	This study
pGPPSmTC/S35	pBbB2a-trAgGPPS(co)- traTerS_Sa	pBBR, Ampr, Ptet, trAgGPPS(co)- traTerS_Sa	This study
pGPPSmTC/S36	pBbB2a-trAgGPPS(co)- trTerS_Ob	pBBR, Ampr, Ptet, trAgGPPS(co)- trTerS_Ob	This study
pGPPSmTC/S37	pBbB2a-trAgGPPS(co)- trTerS_Pm	pBBR, Ampr, Ptet, trAgGPPS(co)- trTerS_Pm	This study

Supporting Results

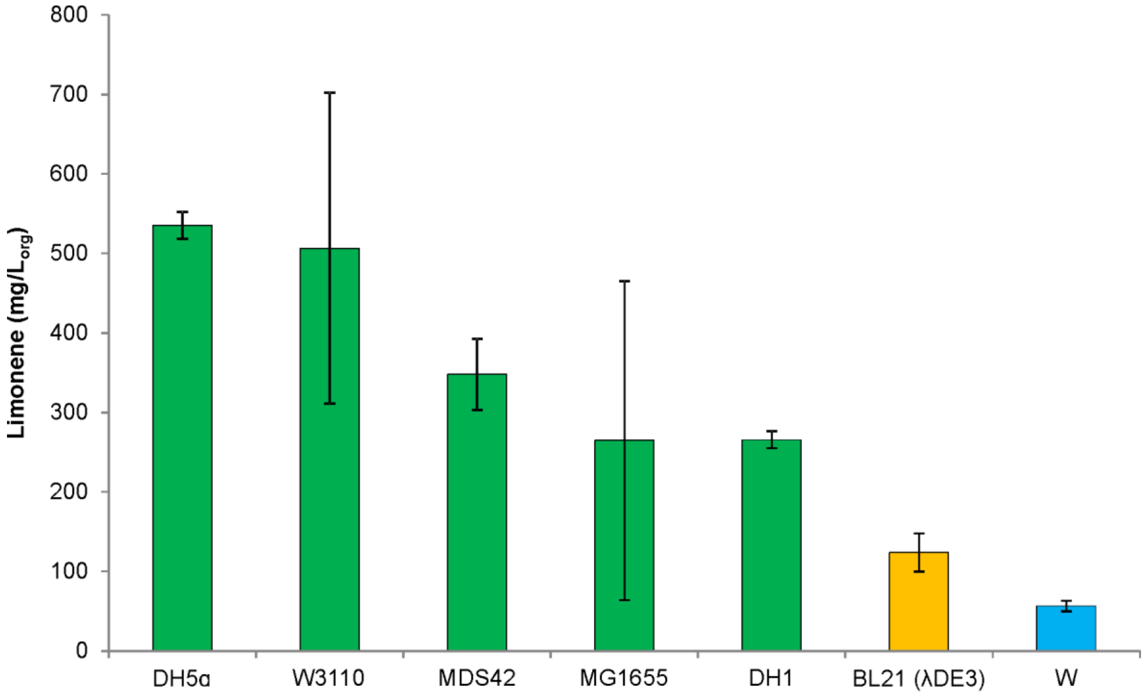

Figure S2: Limonene production titres in different *E. coli* strains. Final titres of limonene produced by *E. coli* strains (Table S1) bearing plasmid pJBEI-6410 after 72 h growth in a bi-phasic culture (concentrations in 20 % organic phase), as calculated from GC analysis. Average titres from triplicate biological repeats; error bars represent standard deviation. Bar colour denotes *E. coli* strain type; K-12 (green), B (orange) or W (blue).

Figure S3: GCMS analysis of authentic monoterpenoid standards.

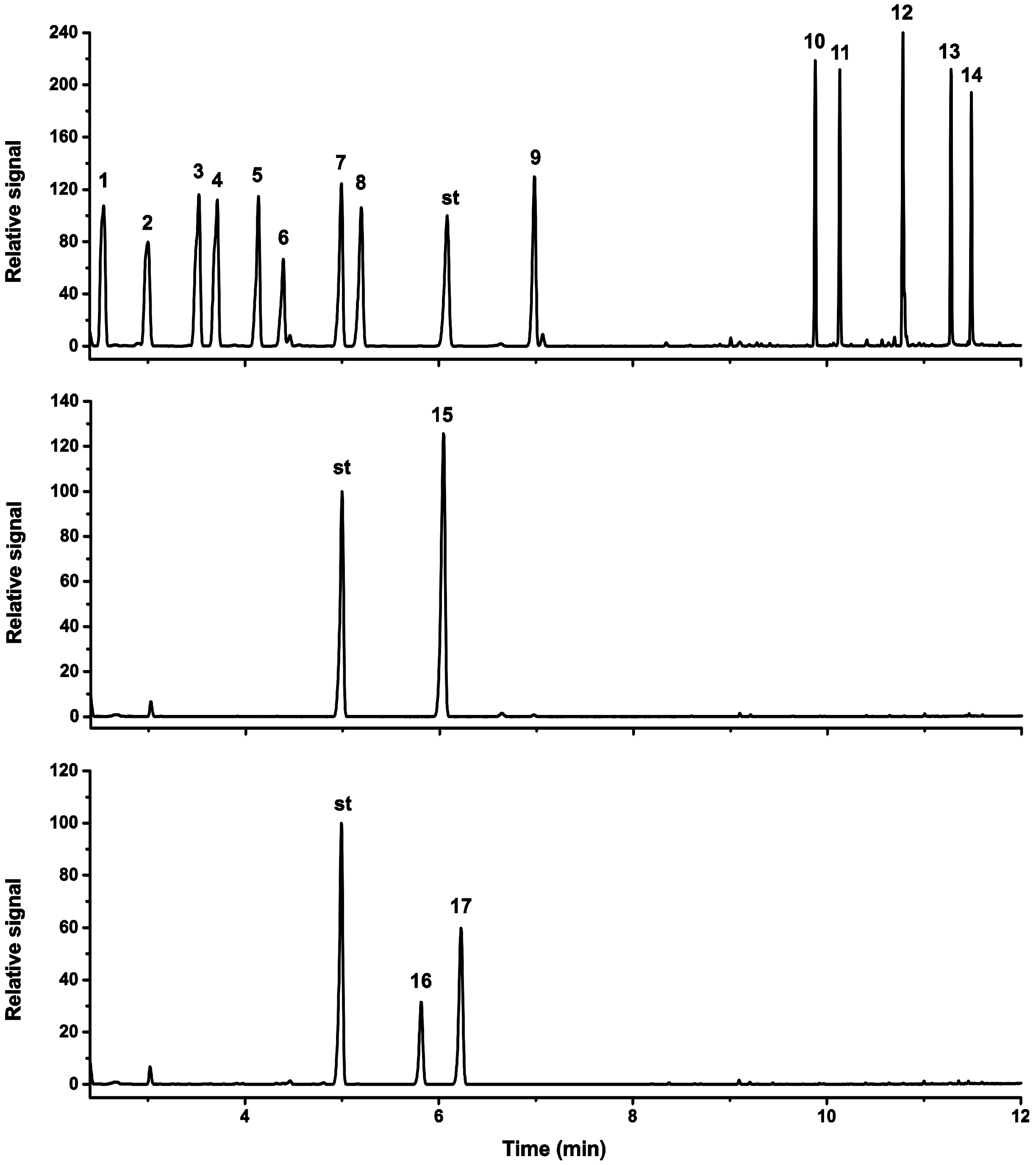

A) GCMS traces of authentic monoterpenoid standards. GCMS traces showing the separation of monoterpenoids (1 mg mL^{-1}) produced in this study on a DB-WAX column. The internal standard used, *sec*-butylbenzene (0.1%, vv), has a retention time of 6.10 minutes. Peak **1**: α-pinene (rt: 2.544), **2**: camphene (rt: 3.001), **3**: β-pinene (rt: 3.525), **4**: sabinene (rt: 3.714), **5**: 3-carene (rt: 4.141), **6**: β-myrcene (rt: 4.395), **7**: limonene (rt: 4.996), **8**: 1,8-cineole (rt: 5.200), **9**: terpinolene (rt: 6.985), **10**: linalool (rt: 9.881), **11**: fenchol (rt: 10.133), **12**: α-terpineol (rt: 10.788), **13**: nerol (rt: 11.208), **14**: geraniol (rt: 11.490). Because γ-terpinene (rt: 6.040) (peak **15**) and (*E*)-β-ocimene (rt: 6.226) (peak **17**), have similar retention times as *sec*-butylbenzene (rt: 6.085), limonene (0.1% vv) was used as internal standard in β-ocimene and γ-terpinene containing samples. The β-ocimene standard contains about 30% (*Z*)-β-ocimene (rt: 5.815) (peak **16**). Method: the injector temperature was set at 240°C with a split ratio of 20:1 (1 μL injection). The carrier gas was helium with a flow rate of 1 mL/min and a pressure of 5.1 psi. The following oven program was used: 50°C (1 min hold), ramp to 68°C at 5°C/min (2 min hold), and ramp to 230°C at 25°C/min (2 min hold). The ion source temperature of the mass spectrometer (MS) was set to 230°C and spectra were recorded from m/z 50 to m/z 250.

B) MS spectra of authentic monoterpenoid standards. Chemical structures are shown as insets.

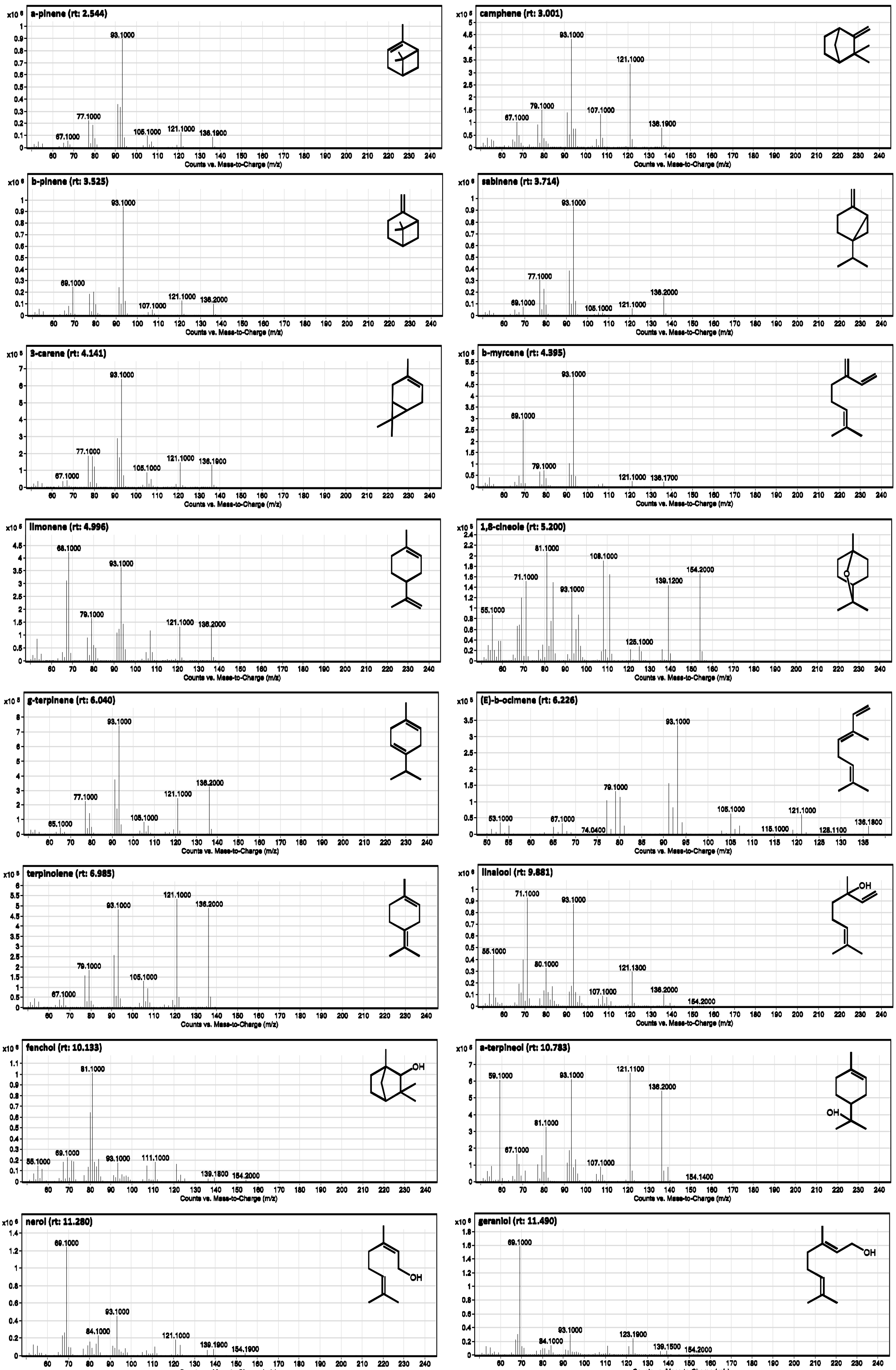

Figure S4: GCMS analysis monoterpenoid production strains. Representative GCMS traces of *n*-nonane overlays obtained from two-phase *E. coli* α-Select cultures containing pMVA and pGPPSmTC/S plasmids are shown. MS spectra of indicated monoterpenoid peaks are shown. Retention times of additional terpenoid peaks are mentioned below. Chemical structures of detected monoterpenoids are shown as insets. Retention times and MS spectra of the detected peaks were compared to retention times and MS spectra of authentic standards wherever possible (See Figure S3). No authentic standards were available for the following monoterpenoids which are produced as by-products: β-phellandrene, 4-carene, δ-terpineol, sabinene hydrate, thujene, borneol, Pinan-2-ol, citronellal, neral, geranial, and citronellol. Also no authentic standards were available for the sesquiterpene by-products farnesal, farnesene and (*E*)-α-bisabolene. Identification of these compounds was achieved by comparing the obtained MS spectra and fragmentation patterns to the NIST reference library.

A) (-)aPinS_Pt

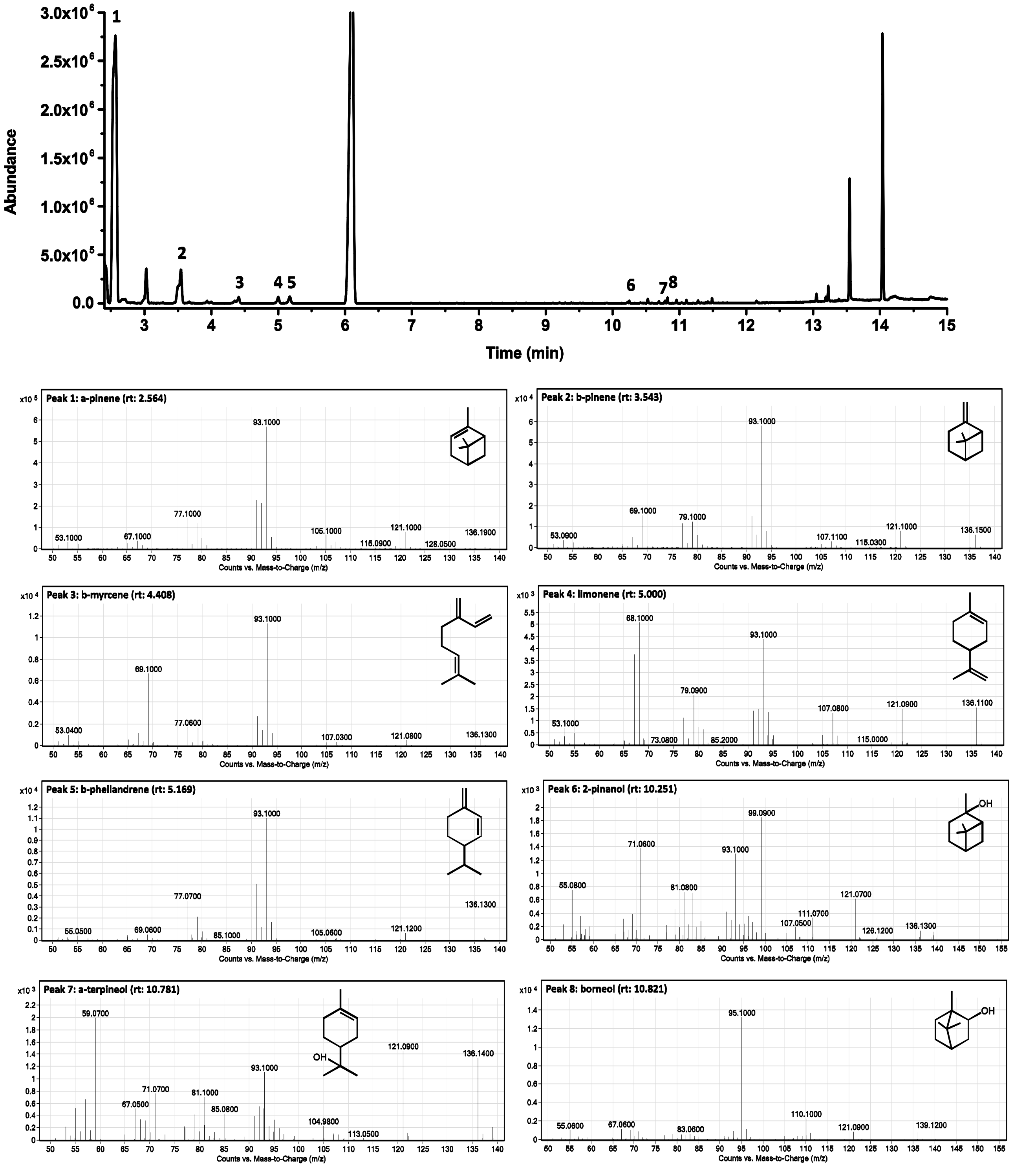

Additional terpenoids detected: geranial (rt: 10.955), citronellol (rt: 11.101), nerol (rt: 11.278), geraniol (rt: 11.487), farnesal (rt: 13.225), and farnesol (rt: 13.542). The peak at rt 14.037 is indole.

B) SLimS_Ms

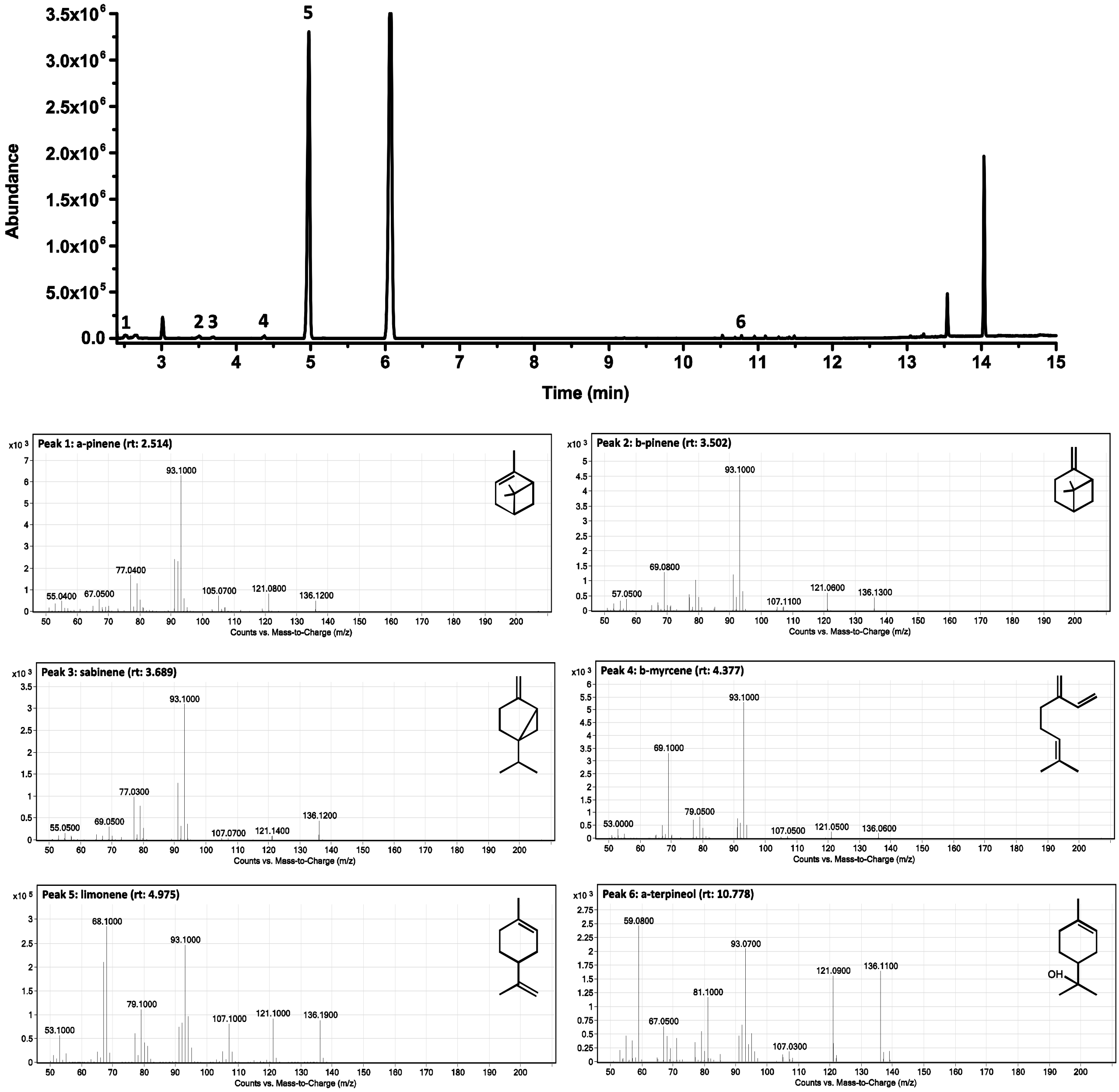

Additional terpenoids detected: geranial (rt: 10.952), citronellol (rt: 11.101), nerol (rt: 11.278), geraniol (rt: 11.487), farnesal (rt: 13.222), and farnesol (rt: 13.540). The peak at rt 14.034 is indole.

C) RLimS_CI

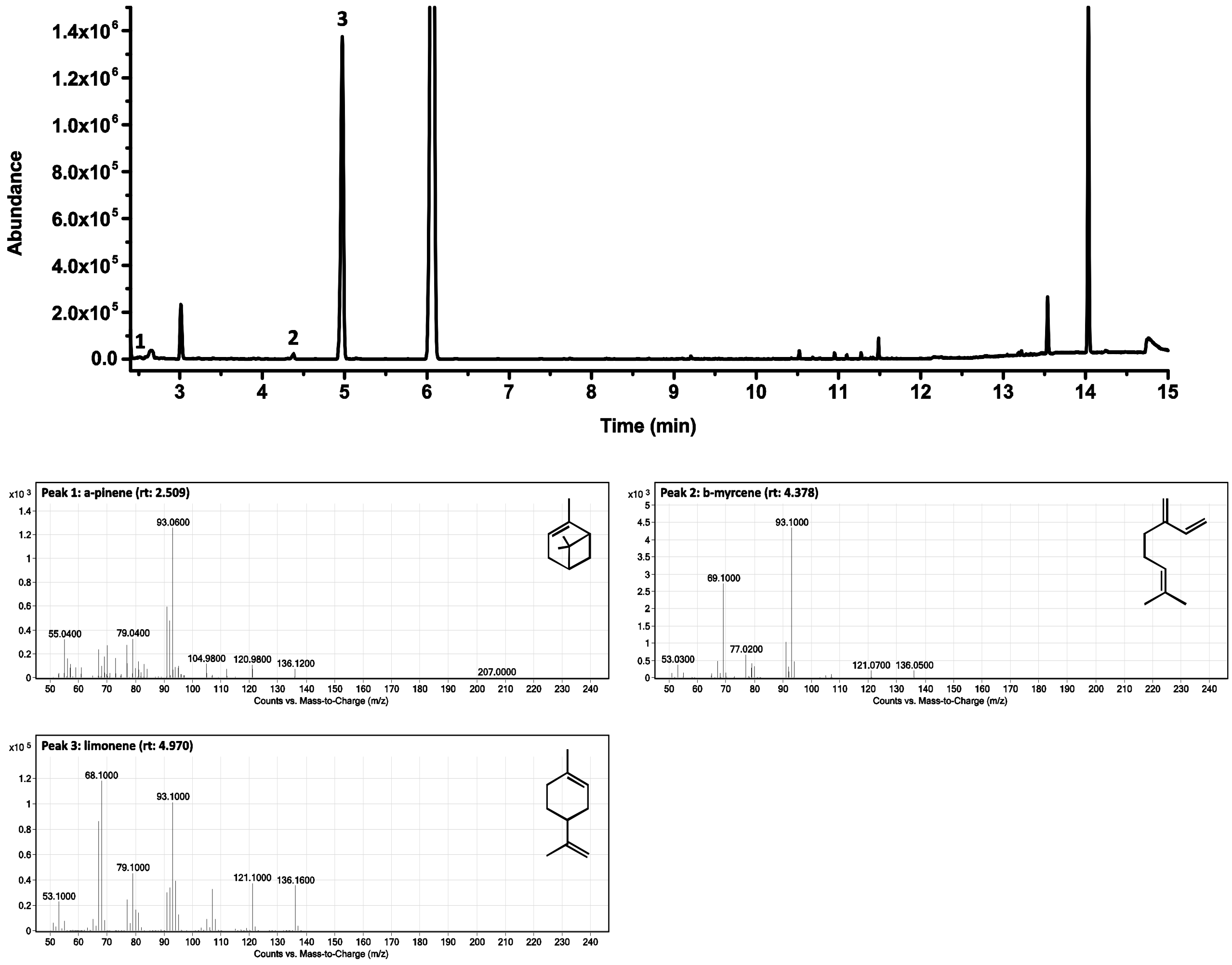

Additional terpenoids detected: geranial (rt: 10.952), citronellol (rt: 11.099), nerol (rt: 11.275), geraniol (rt: 11.484), farnesal (rt: 13.222), and farnesol (rt: 13.540). The peak at rt 14.034 is indole.

D) gTerS_Ov

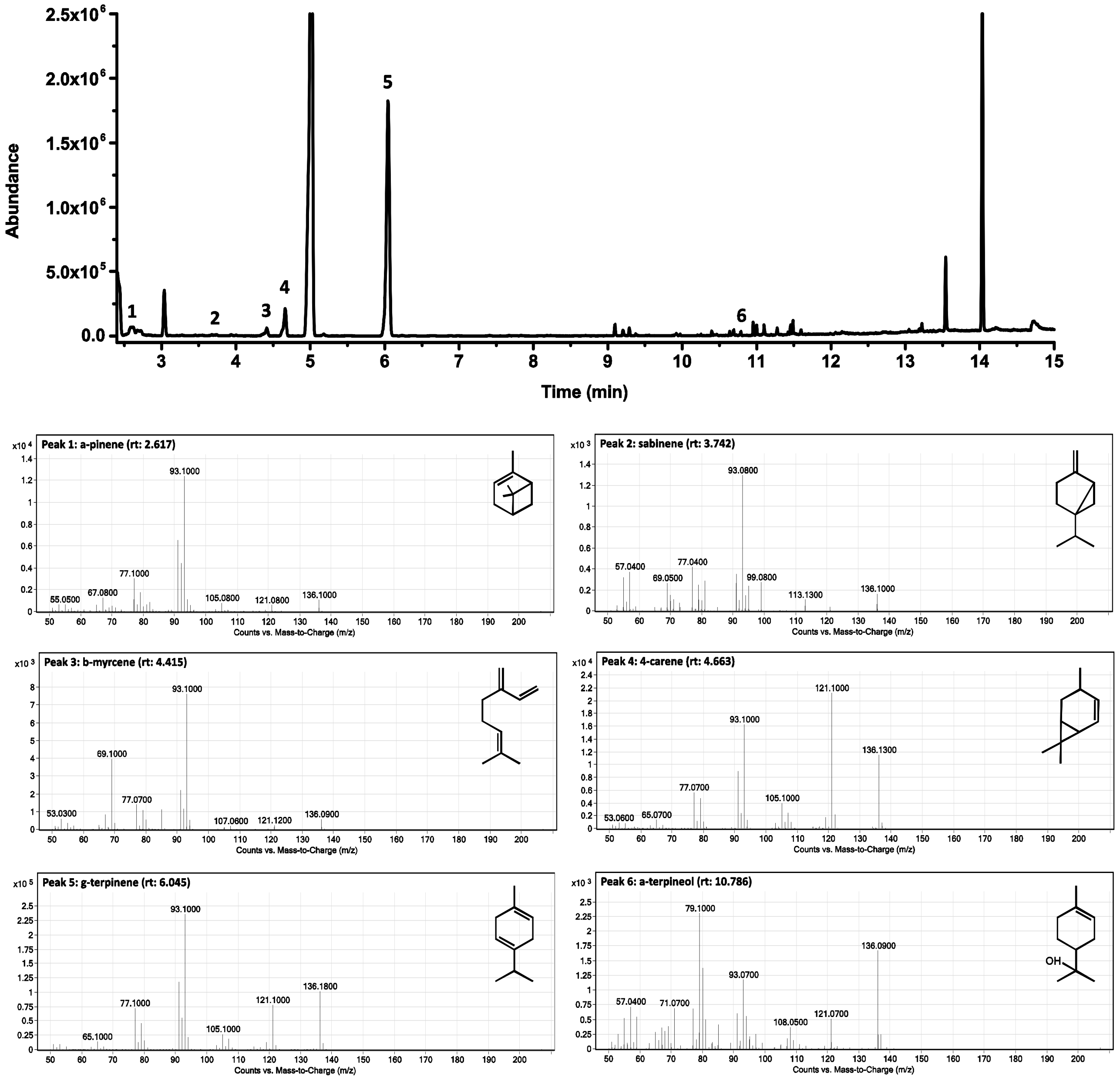

Additional terpenoids detected: citronellol (rt: 11.101), nerol (rt: 11.278), geraniol (rt: 11.489), farnesal (rt: 13.222), and farnesol (rt: 13.542). The peak at rt 14.034 is indole.

E) (+)aPinS_Pt

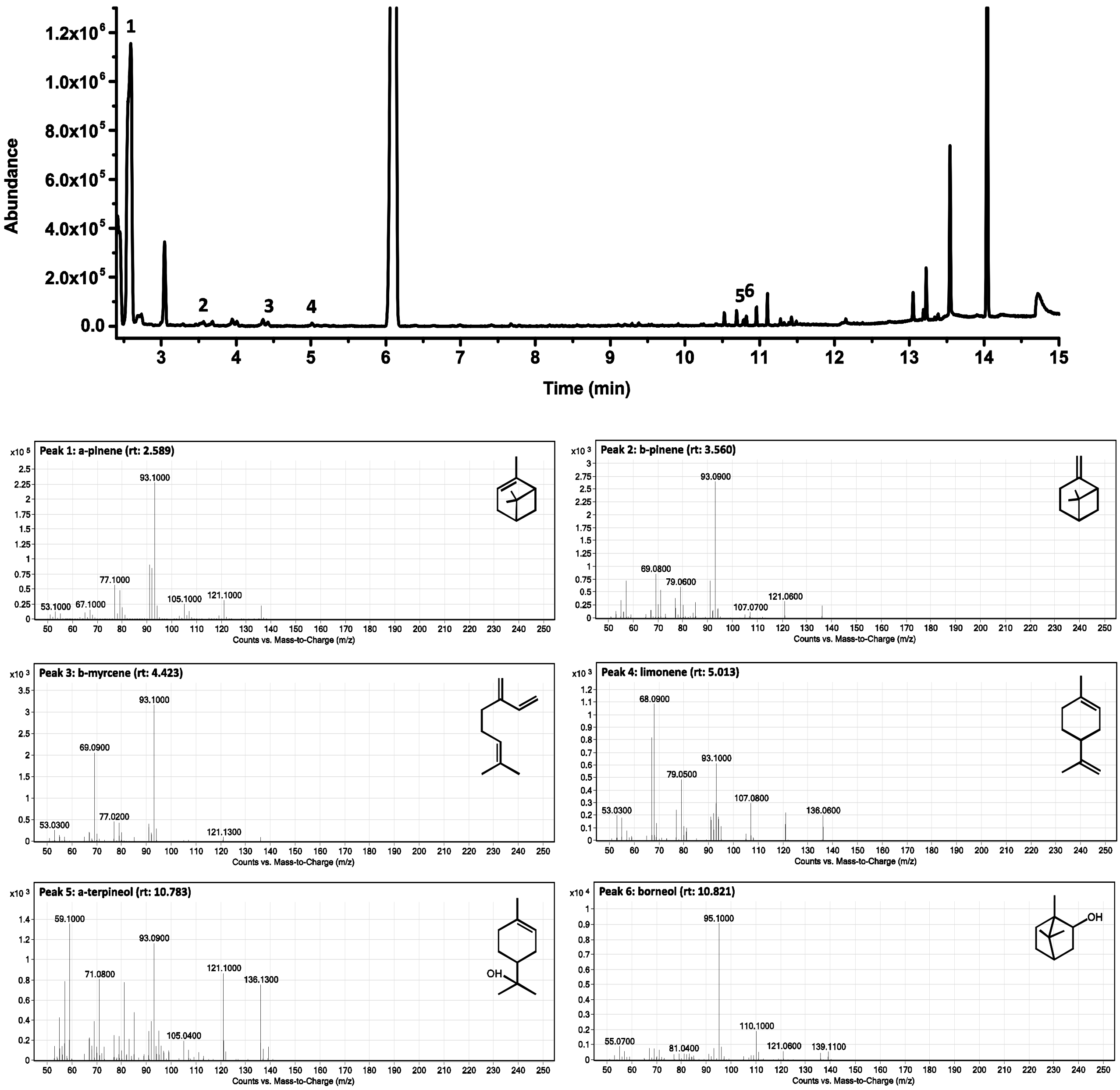

Additional terpenoids detected: neral (rt: 10.690), geranial (rt: 10.957), citronellol (rt: 11.101), nerol (rt: 11.278), geraniol (rt: 11.490), and farnesol (rt: 13.542). The peak at rt 14.039 is indole.

F) CinS_Sf

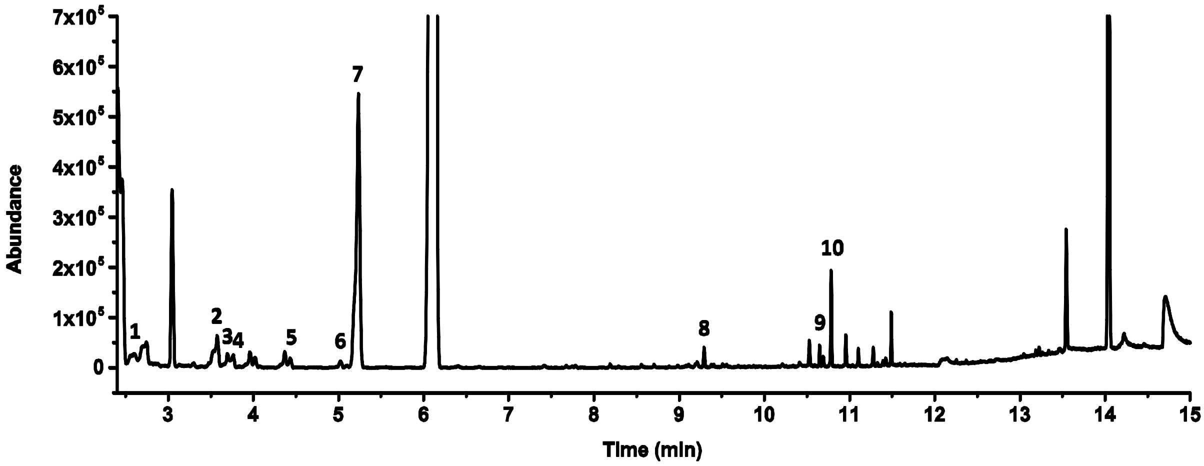

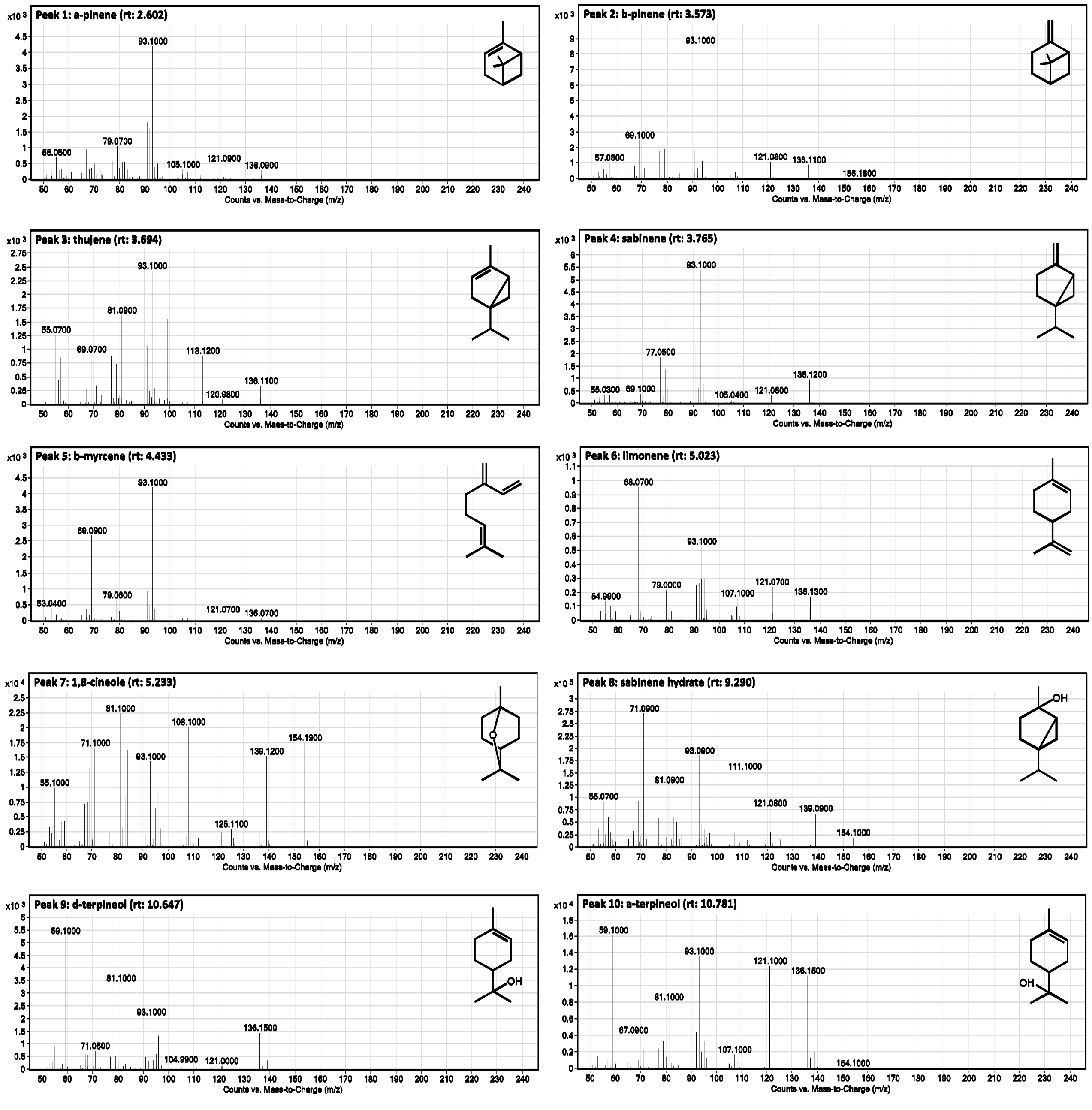

Additional terpenoids detected: neral (rt: 10.690), geranial (rt: 10.955), citronellol (rt: 11.101), nerol (rt: 11.278), geraniol (rt: 11.487), and farnesol (rt: 13.542). The peak at rt 14.039 is indole.

G) (-)bPinS_Aa

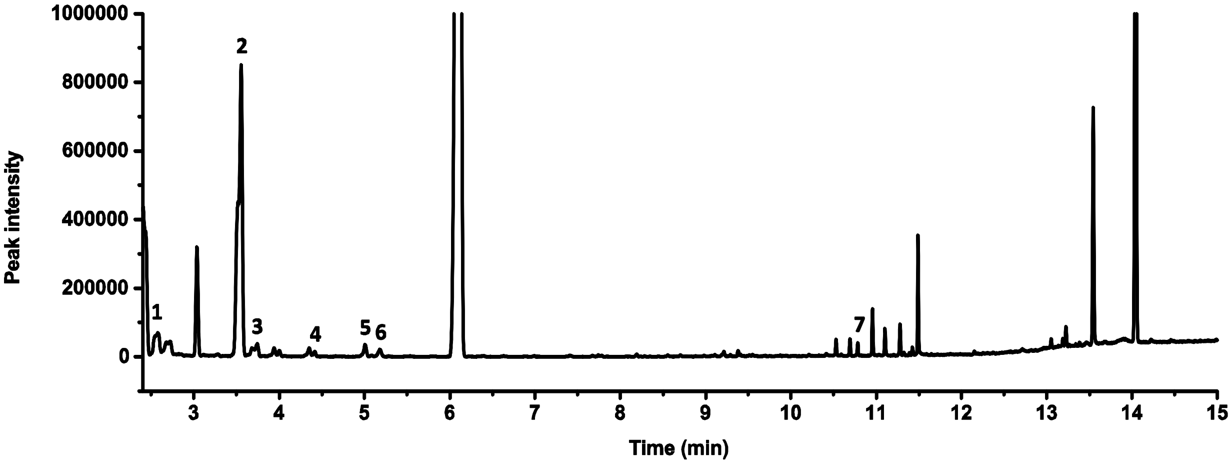

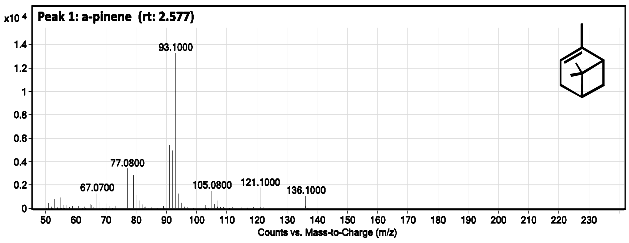

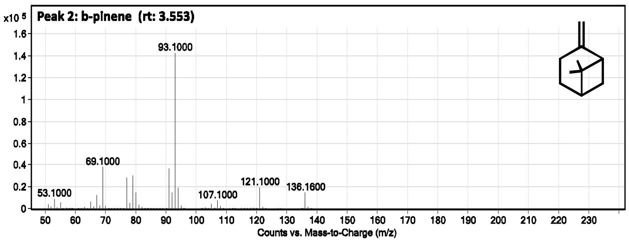

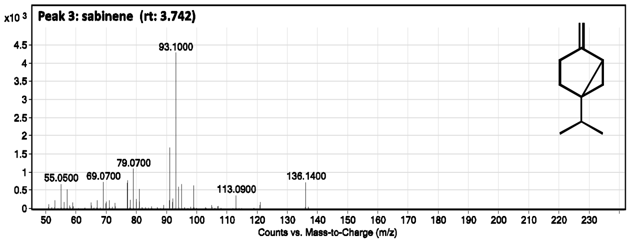

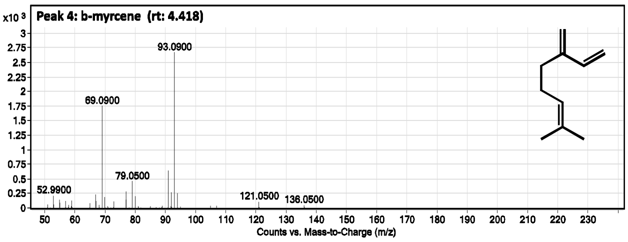

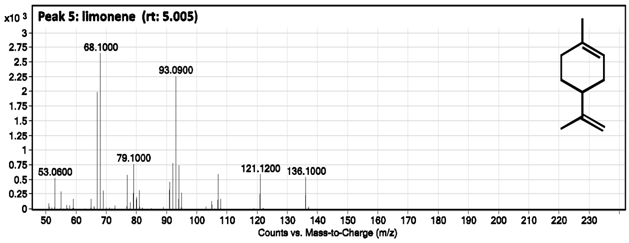

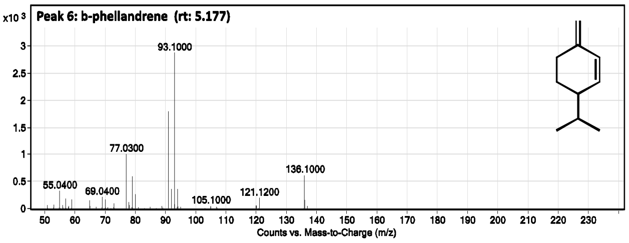

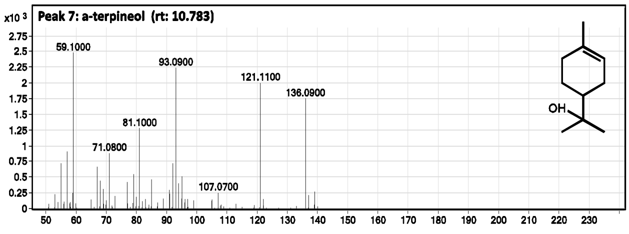

Additional terpenoids detected: neral (rt: 10.690), geranial (rt: 10.957), citronellol (rt: 11.101), nerol (rt: 11.278), geraniol (rt: 11.487), and farnesol (rt: 13.542). The peak at rt 14.039 is indole.

H) GerS_Pc

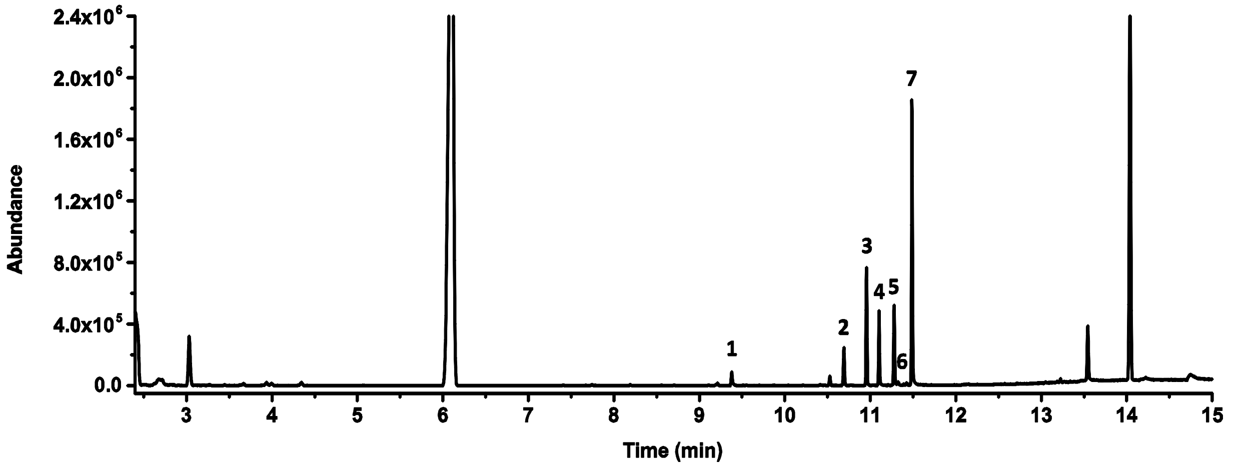

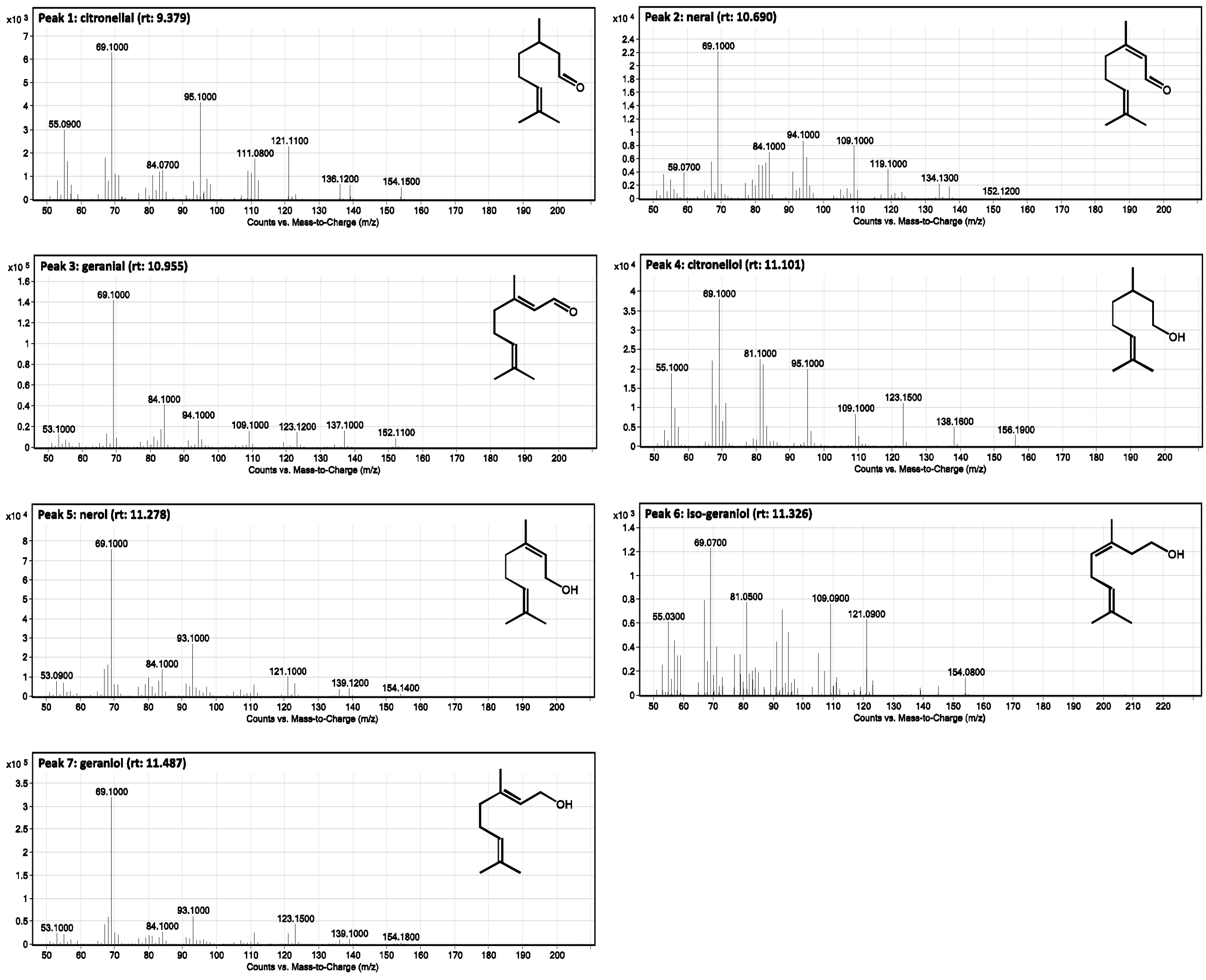

Additional terpenoids detected: farnesol (rt: 13.542). The peak at rt 14.037 is indole.

I) CinS_At

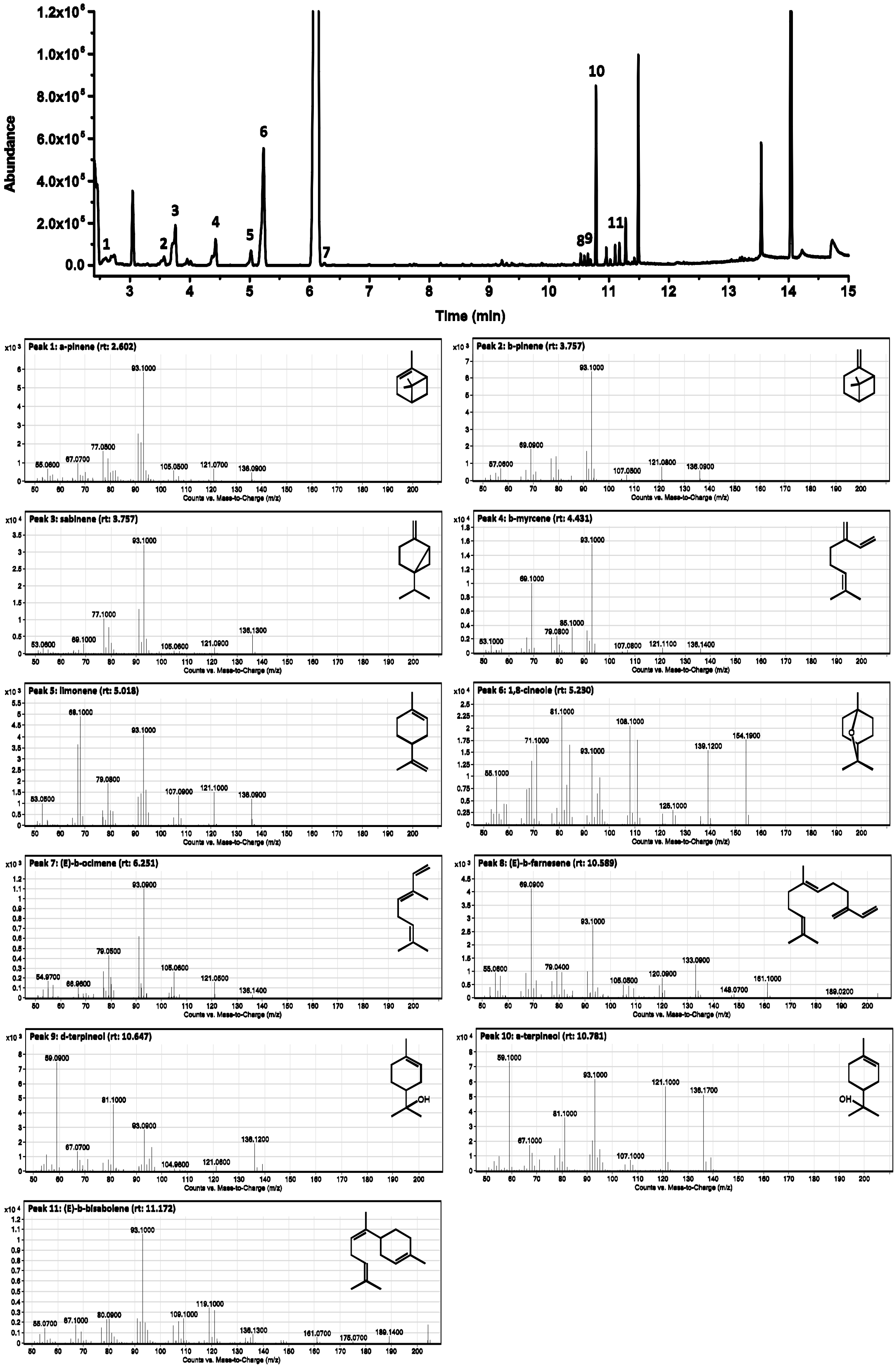

Additional terpenoids detected: geranial (rt: 10.955), citronellol (rt: 11.101), nerol (rt: 11.278), geraniol (rt: 11.487), and farnesol (rt: 13.540). The peak at rt 14.037 is indole.

J) FenS_Lv

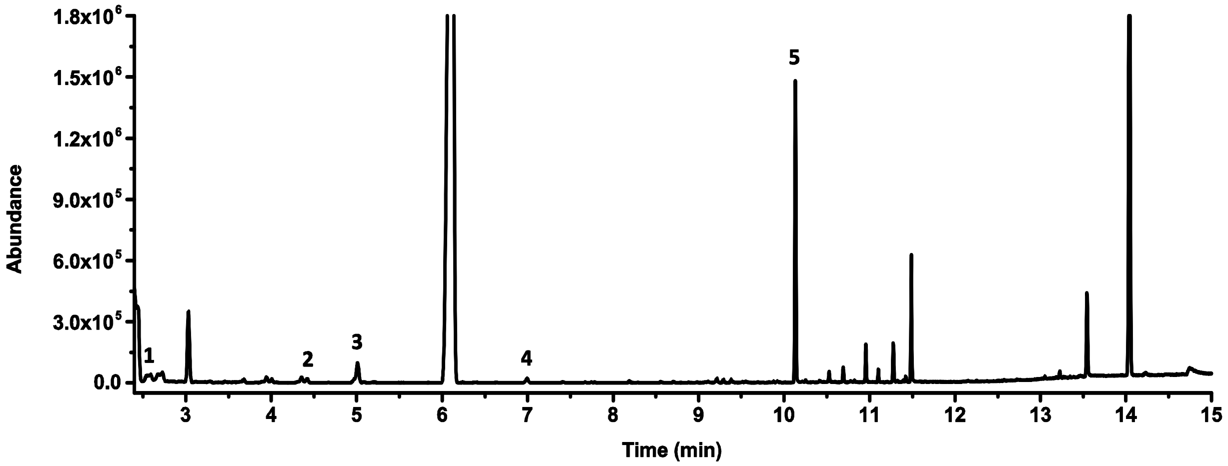

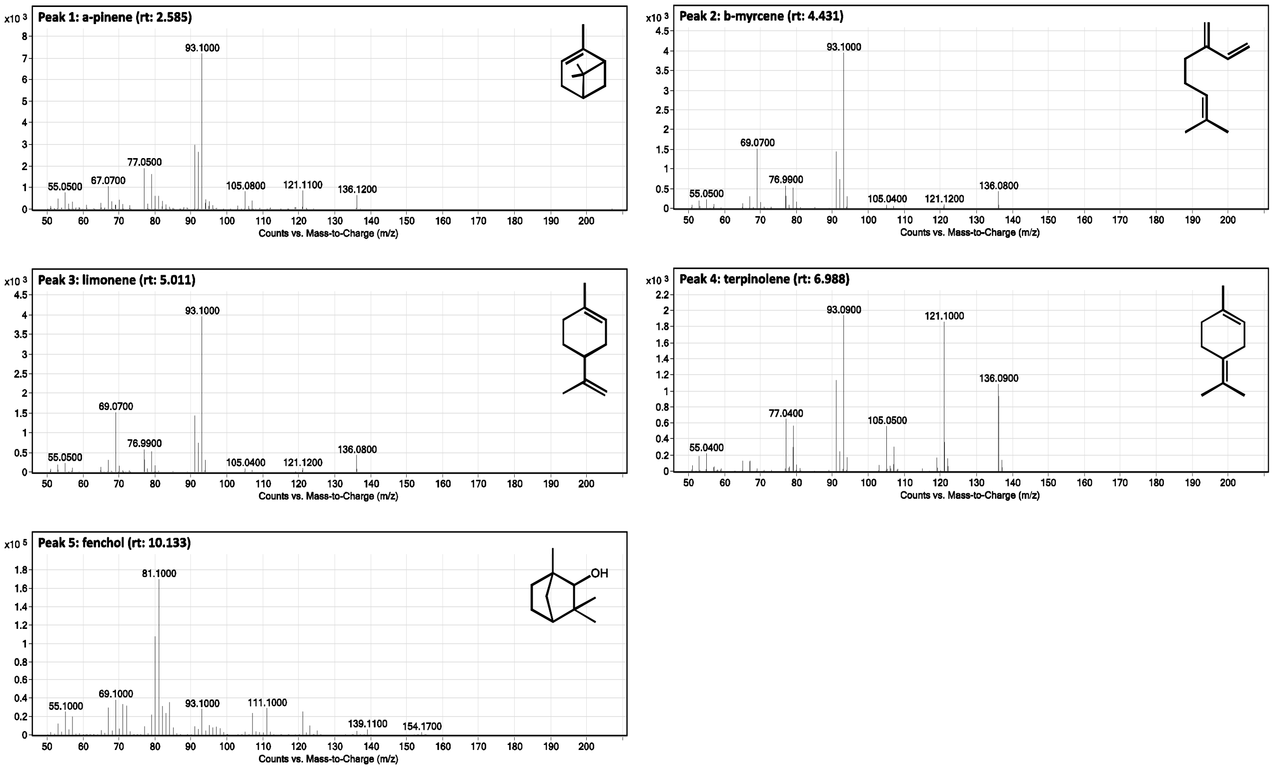

Additional terpenoids detected: citronellal (rt: 9.376), neral (rt: 10.690), geranial (rt: 10.957), citronellol (rt: 11.101), nerol (rt: 11.278), geraniol (rt: 11.487), and farnesol (rt: 13.543). The peak at rt 14.039 is indole.

K) SabS_Sp

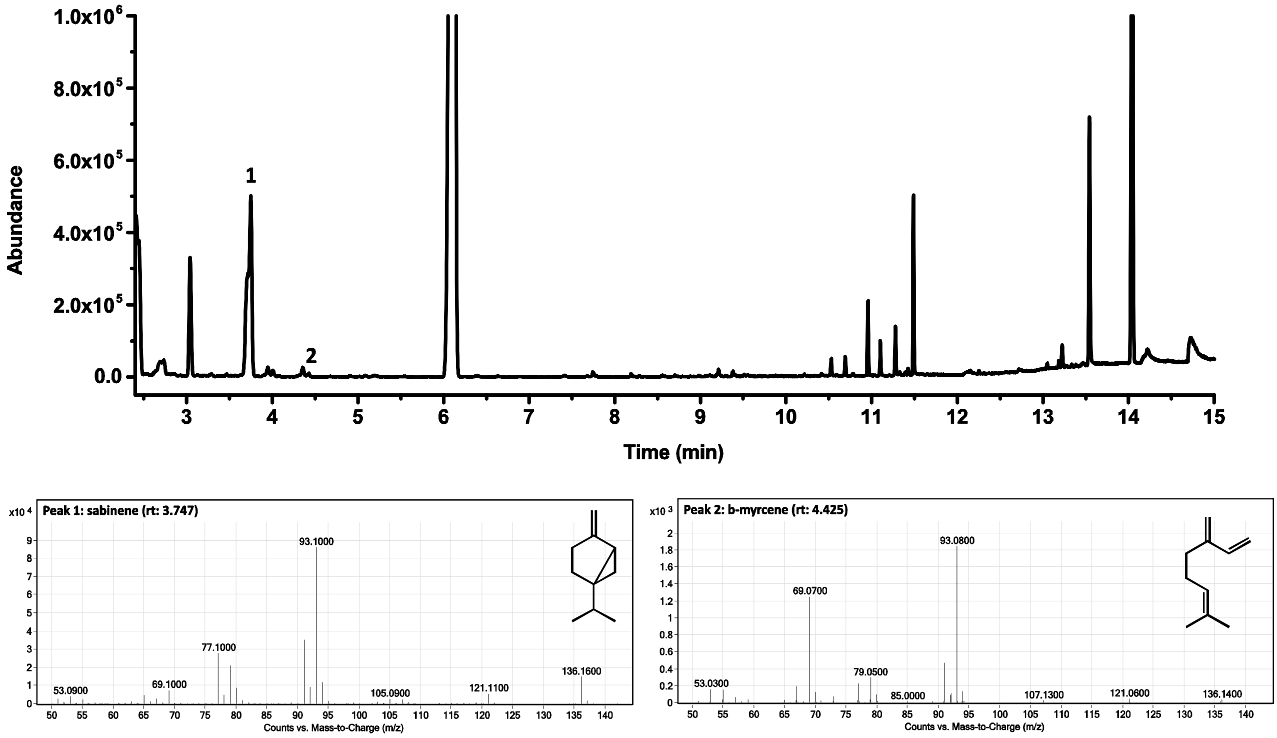

Additional terpenoids detected: neral (rt: 10.690), geranial (rt: 10.959), citronellol (rt: 11.101), nerol (rt: 11.278), geraniol (rt: 11.489), farnesal (rt: 13.224), and farnesol (rt: 13.543). The peak at rt 14.039 is indole.

L) aTerS_Mg

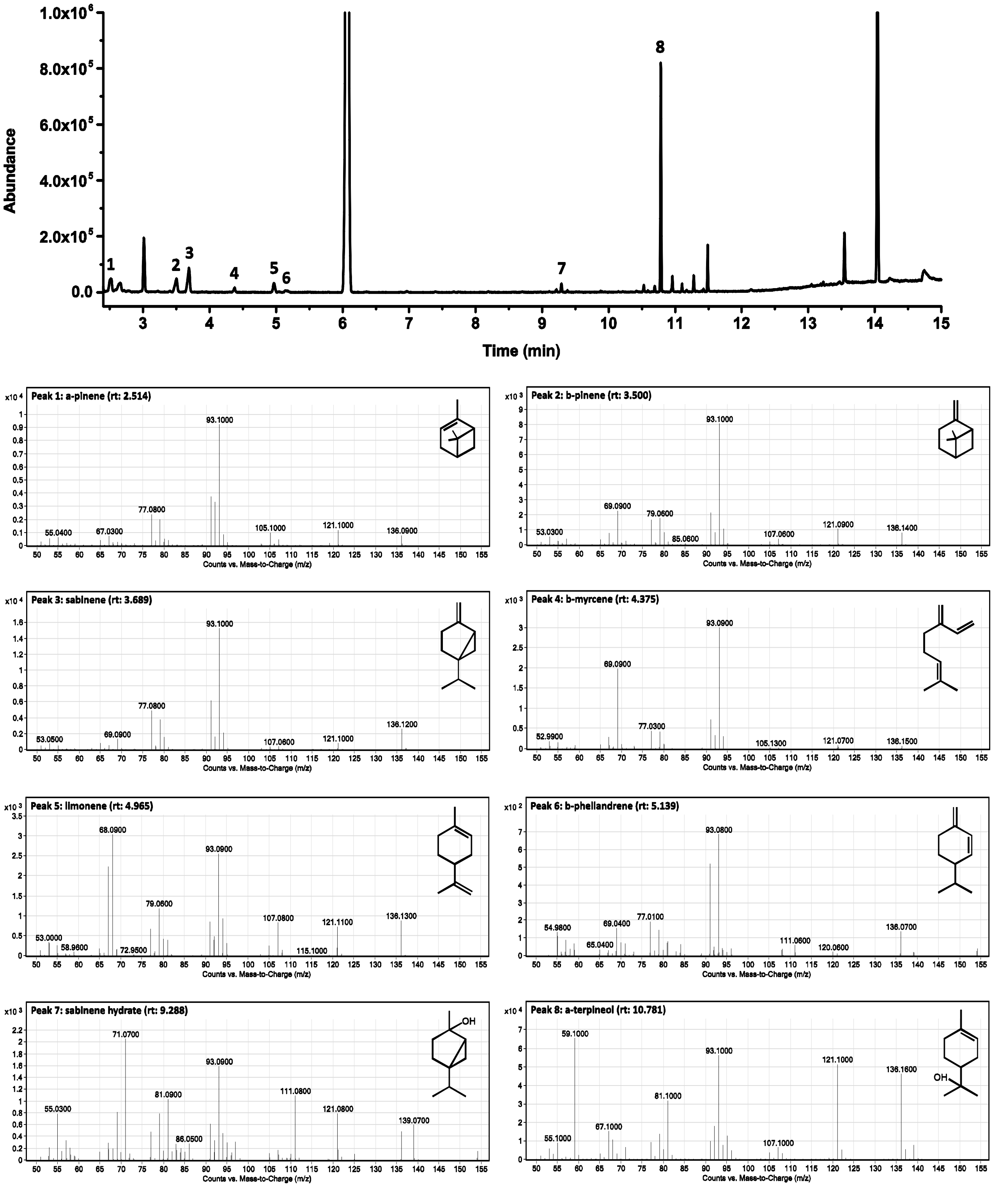

Additional terpenoids detected: neral (rt: 10.690), geranial (rt: 10.957), citronellol (rt: 11.101), nerol (rt: 11.278), geraniol (rt: 11.487), and farnesol (rt: 13.542). The peak at rt 14.039 is indole

M) OciS_Am

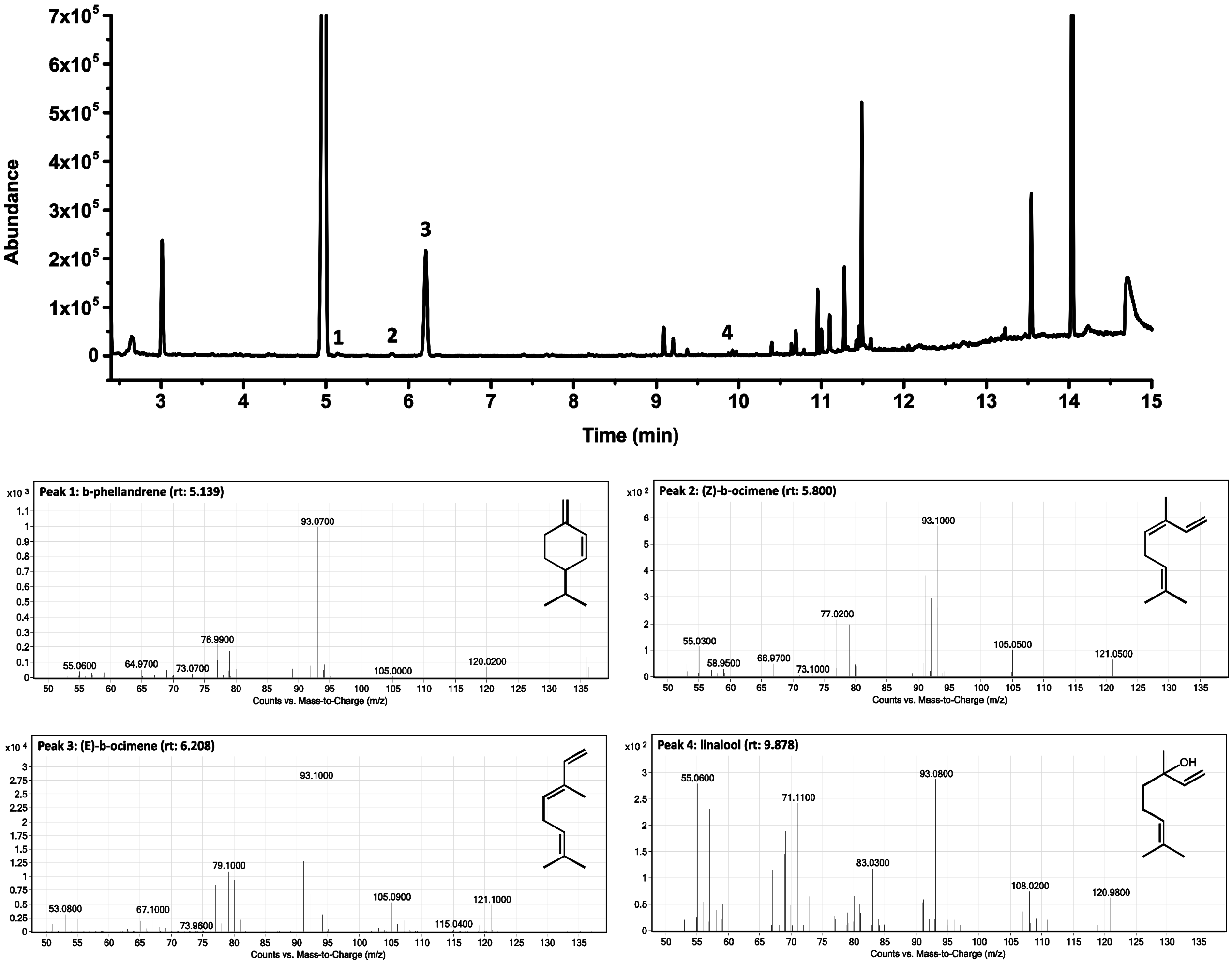

Additional terpenoids detected: neral (rt: 10.690), geranial (rt: 10.955), citronellol (rt: 11.101), nerol (rt: 11.278), geraniol (rt: 11.487), and farnesol (rt: 13.542). The peak at rt 14.037 is indole.

N) CamS_SI

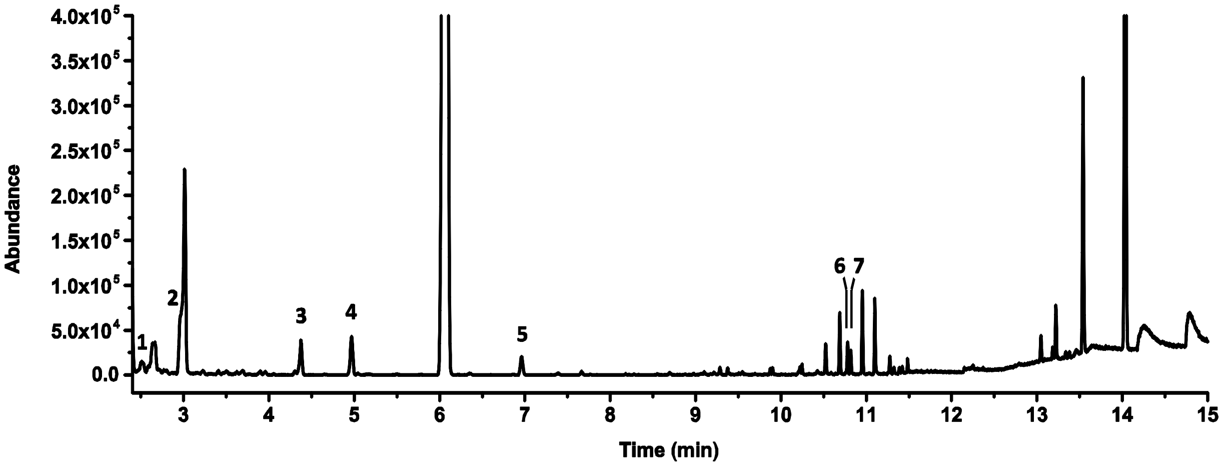

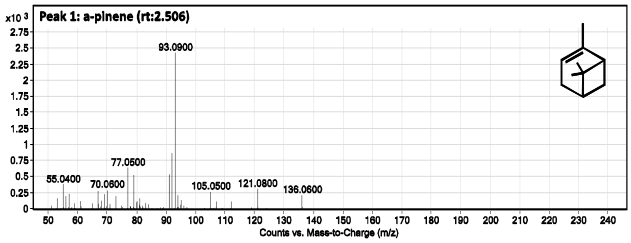

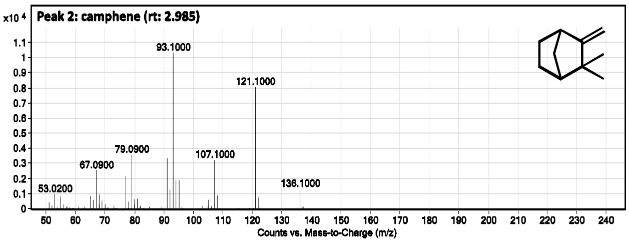

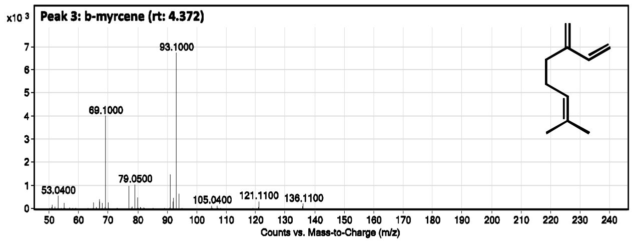

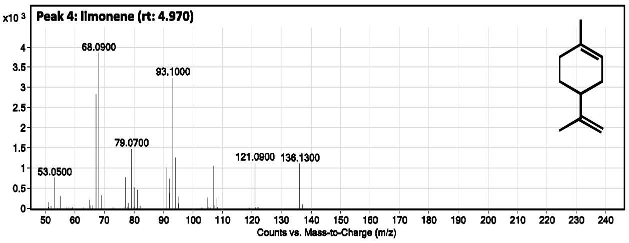

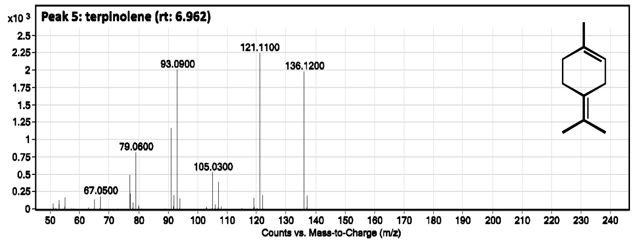

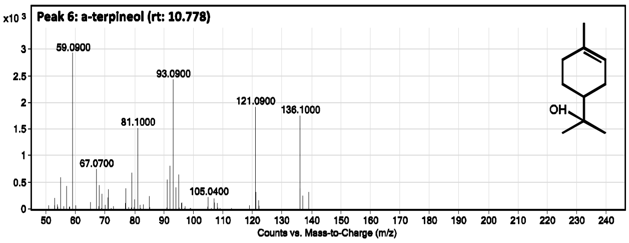

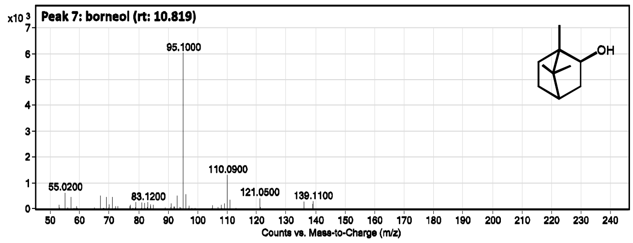

Additional terpenoids detected: neral (rt: 10.687), geranial (rt: 10.955), citronellol (rt: 11.098), nerol (rt: 11.275), geraniol (rt: 11.484), and farnesol (rt: 13.540). The peak at rt 14.034 is indole.

O) CinS_Cu

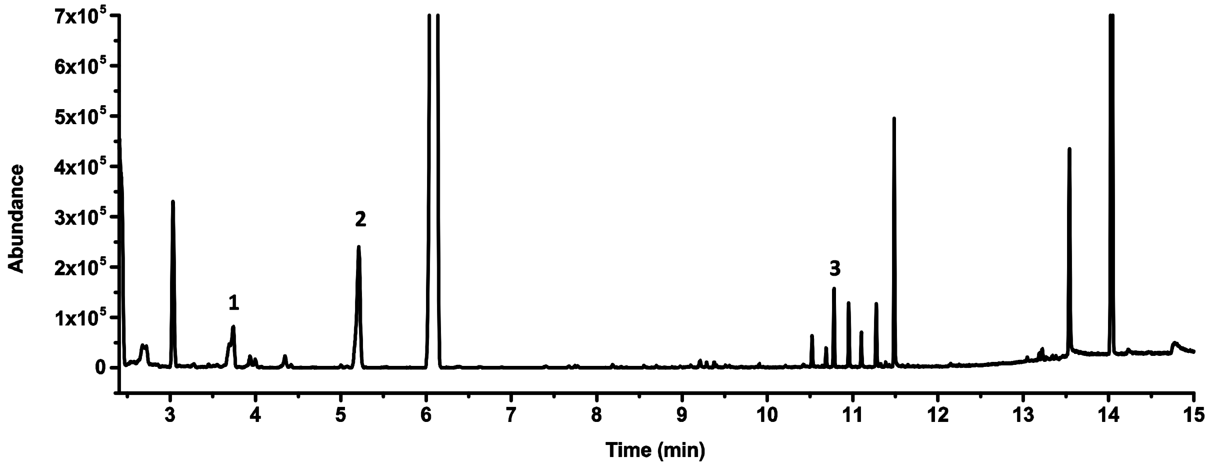

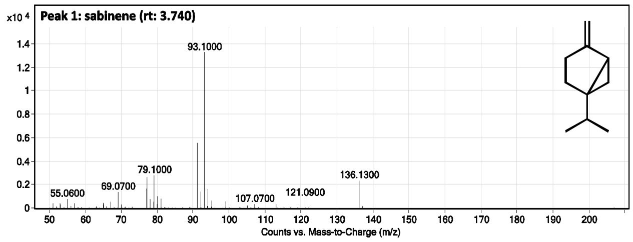

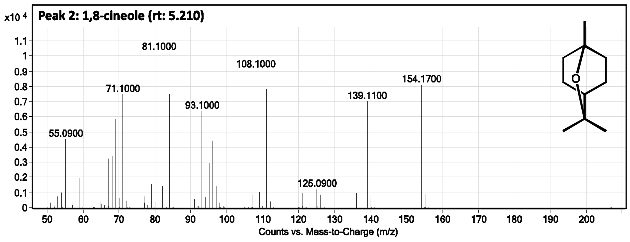

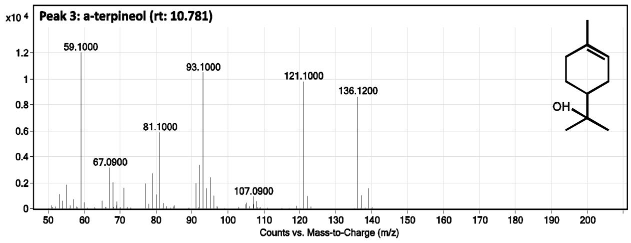

Additional terpenoids detected: neral (rt: 10.688), geranial (rt: 10.955), citronellol (rt: 11.101), nerol (rt: 11.278), geraniol (rt: 11.487), and farnesol (rt: 13.540). The peak at rt 14.034 is indole.

P) (-)aPinS_Ps

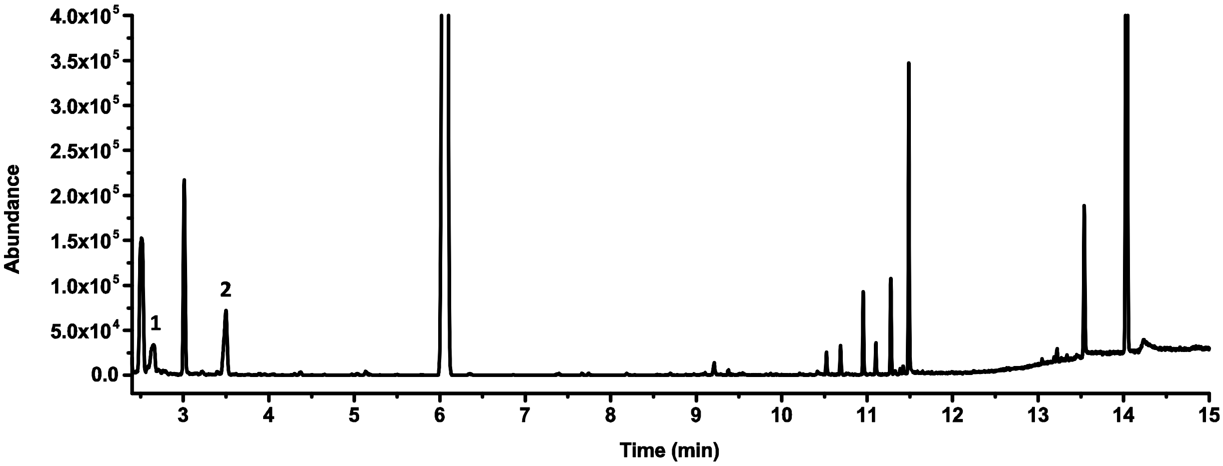

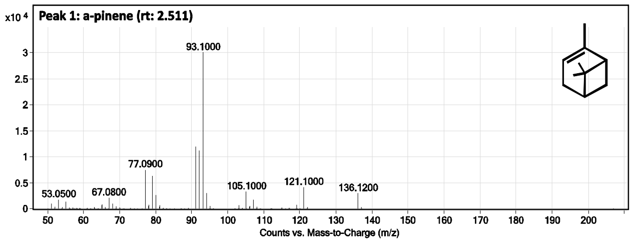

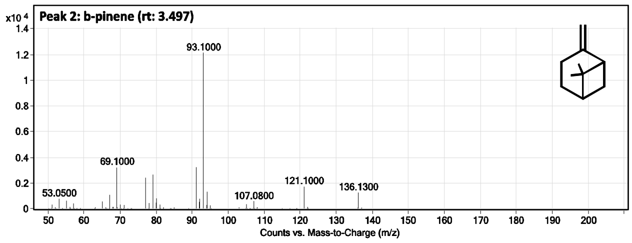

Additional terpenoids detected: neral (rt: 10.688), geranial (rt: 10.955), citronellol (rt: 11.101), nerol (rt: 11.278), geraniol (rt: 11.487), and farnesol (rt: 13.540). The peak at rt 14.034 is indole.

Q) CarS_Pa

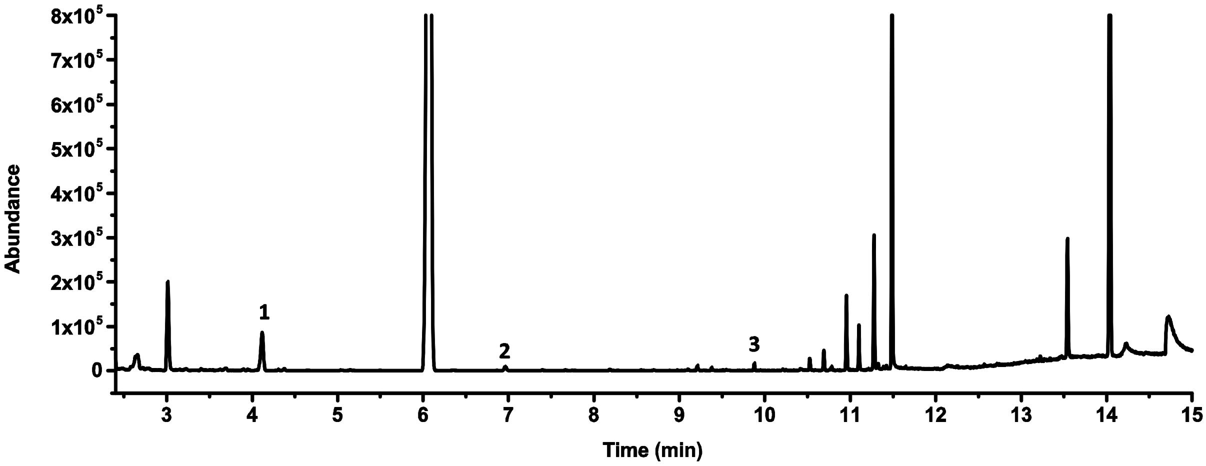

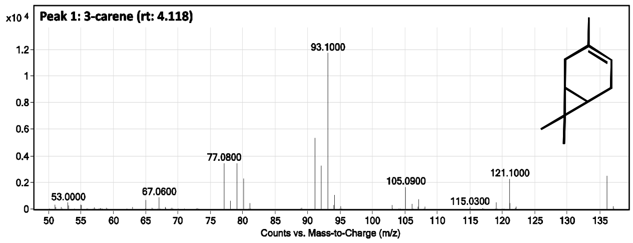

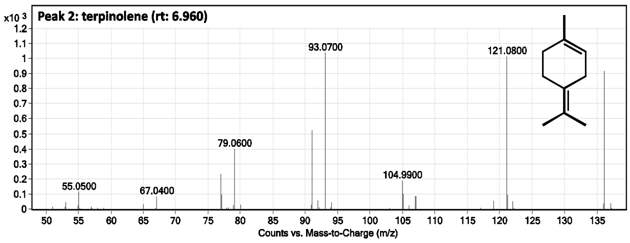

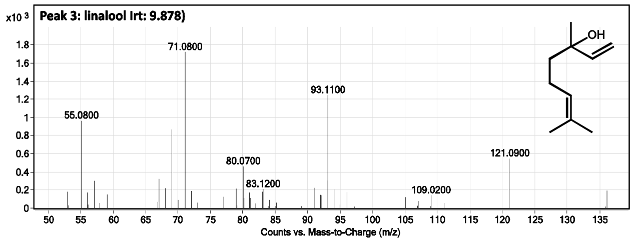

Additional terpenoids detected: neral (rt: 10.690), geranial (rt: 10.955), citronellol (rt: 11.101), nerol (rt: 11.278), geraniol (rt: 11.487), and farnesol (rt: 13.542). The peak at rt 14.037 is indole.

R) GerS_Ob

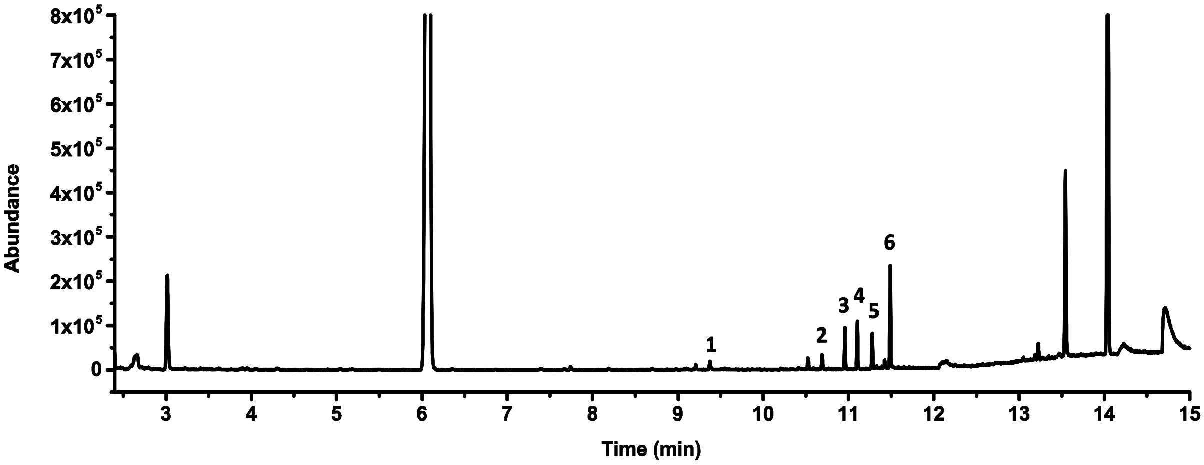

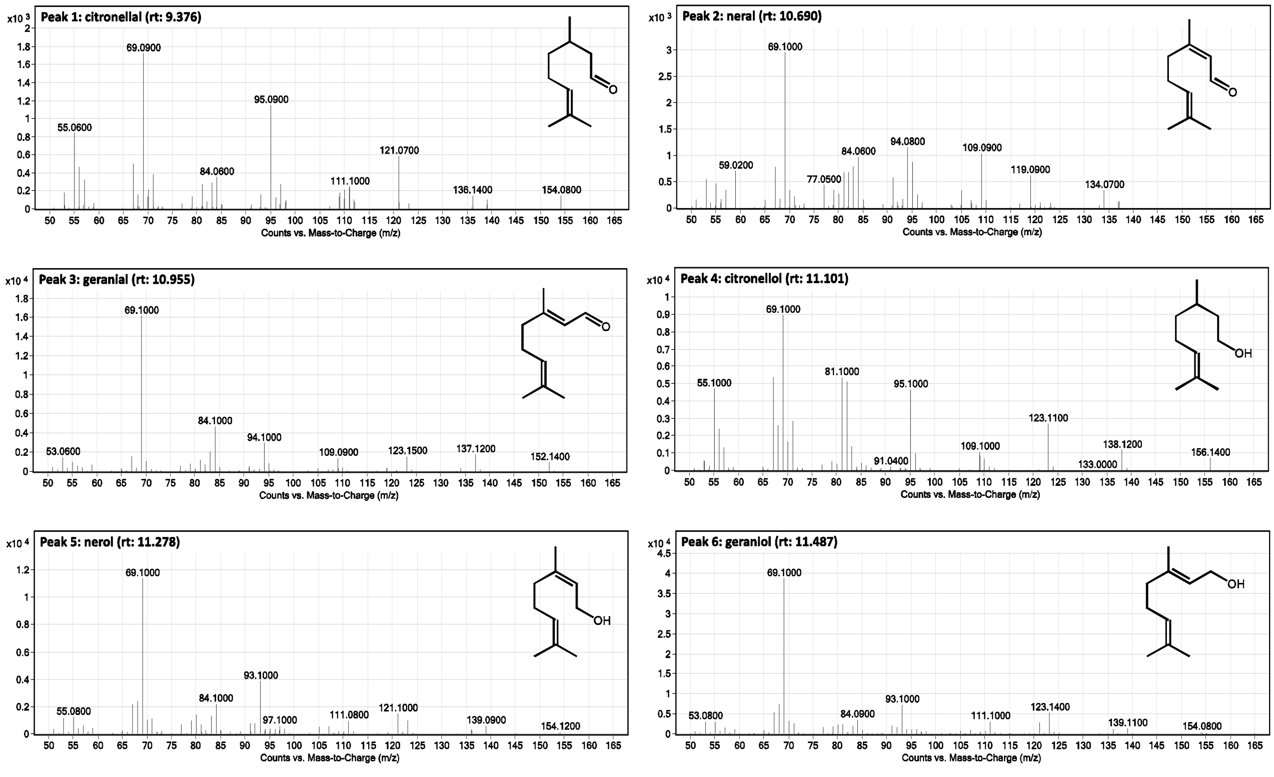

Additional terpenoids detected: farnesal (rt: 13.222), and farnesol (rt: 13.543). The peak at rt 14.039 is indole.

S) GerS_Ct

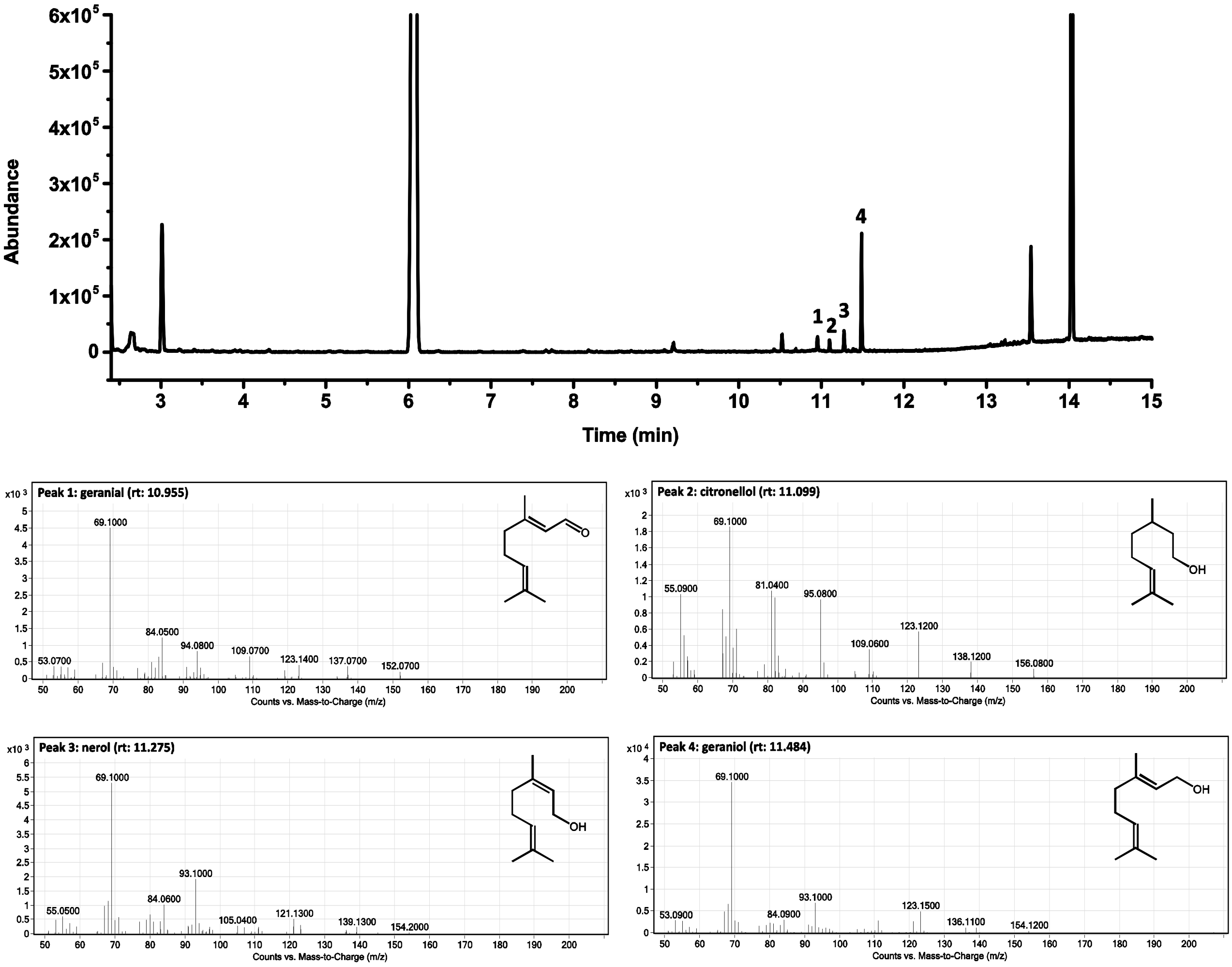

Additional terpenoids detected: farnesol (rt: 13.537). The peak at rt 14.032 is indole.

T) RLinS_Aa

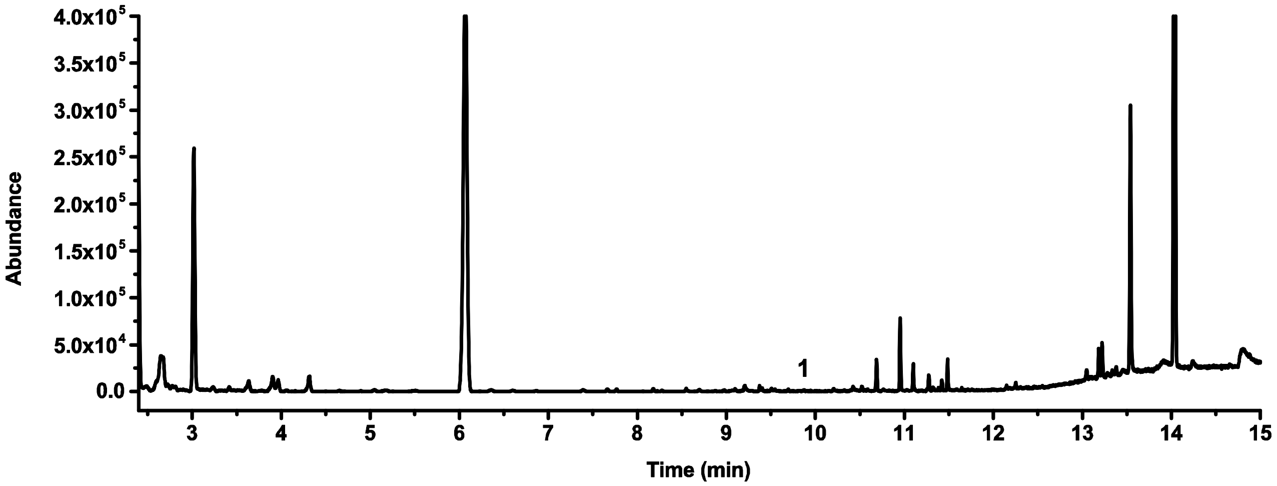

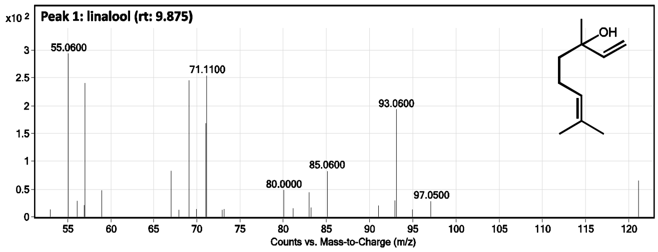

Additional terpenoids detected: neral (rt: 10.687), geranial (rt: 10.952), citronellol (rt: 11.099), nerol (rt: 11.275), geraniol (rt: 11.484), and farnesol (rt: 13.540). The peak at rt 14.034 is indole.

Table S5: Product profiles and monoterpenoid titres of production strains. Product profiles and monoterpenoid titres (mg L_{org}^{-1}) are determined from two-phase cultures with an *n*-nonane overlay for each *E. coli* strain containing the MVA pathway and a distinct monoterpene synthase. Averages of at least 3 biological replicates per mTC/S and the corresponding standard deviations (in brackets) are shown. Highest values for each compound are highlighted in bold.

	**Linear monoterpenoids**				**Mono-cyclic monoterpenoids**					**Bi-cyclic monoterpenoids**										
**Synthase**	**geraniol**	***(E)*-β-ocimene**	**β-myrcene**	**linalool**	**limonene**	**γ-terpinene**	**α-terpineol**	**β-phellandrene**	**terpinolene**	**α-pinene**	**β-pinene**	**fenchol**	**1,8-cineole**	**sabinene**	**3-carene**	**camphene**	**other minor**	**Total product**	**Geranoids**	**Farnesol**
(-)aPinS_Pt			11.2 (5.6)		6.6 (1.6)		<1	**7.4** (3.5)		**517.2** (132.7)	94.3 (43.1)						3.8 (3.3)	**642**	8.1 (2.4)	41.9 (22.3)
SLimS_Ms			7.7 (2.6)		**530.6** (102.3)		2.1 (0.5)			5.3 (2.3)	5.7 (1.3)			2.3 (0.8)				**554**	7.2 (4.6)	33.2 (11.5)
RLimS_Cl			4.3 (4.4)		260.0 (122.2)					<1								**265**	4.4 (5.5)	17.9 (3.3)
gTerS_Ov			3.1 (3.7)			**197.1** (119.7)	<1			13.3 (6.1)				<1			18.5 (12.7)	**232**	19.2 (15.7)	42.9 (42.3)
(+)aPinS_Pt			3.2 (0.4)		<1		<1			190.6 (31.9)	2.5 (0.5)						<1	**197**	35.2 (12.2)	40.8 (6.1)
CinS_Sf			3.2 (4.5)		1.9 (2.6)		11.4 (6.3)			7.1 (8.7)	33.5 (39.0)		**118.2** (153.6)	3.7 (6.7)			4.0 (3.8)	**183**	12.3 (8.0)	25.9 (33.2)
(-)bPinS_Aa			3.0 (0.4)		2.3 (1.9)		2.1 (0.1)	2.8 (0.4)		12.1 (4.7)	**145.0** (33.5)			6.9 (1.9)				**174**	48.0 (11.9)	41.1 (7.0)
GerS_Pc	**162.6** (98.2)																	**163**	189.9 (147.4)	30.7 (15.6)
CinS_At		<1	**17.3** (15.5)		4.4 (3.3)		20.7 (14.6)			1.9 (3.3)	3.8 (2.7)		46.6 (32.7)	15.2 (13.1)			<1	**111**	47.4 (36.3)	16.6 (7.4)
FenS_Lv			2.7 (2.5)		6.1 (6.1)				1.6 (0.6)	3.1 (1.9)		**68.6** (30.9)						**82.1**	79.0 (7.8)	17.6 (1.4)
SabS_Sp			1.3 (1.5)											**76.6** (74.0)				**77.9**	34.5 (27.5)	19.2 (12.0)
aTerS_Mg			1.9 (3.3)		3.2 (3.4)		**37.9** (36.3)	<1		4.9 (5.4)	6.2 (6.5)			9.2 (9.3)			1.6 (1.6)	**65.5**	28.8 (23.5)	13.2 (7.3)
OciS_Am		**57.3** (56.9)		<1				<1									<1	**59.4**	105.9 (17.6)	19.5 (9.3)
CamS_Sl			10.3 (2.6)		7.4 (0.9)		2.7 (0.2)		**3.4** (0.5)	2.2 (0.4)						**12.2** (3.4)	1.8 (0.1)	**40.0**	42.3 (12.1)	19.3 (0.8)
CinS_Cu							3.6 (4.1)						18.2 (18.3)	7.1 (7.2)				**28.9**	37.7 (33.9)	10.3 (10.6)
(-)aPinS_Ps										18.8 (15.5)	9.9 (7.9)							**28.7**	24.7 (37.0)	8.6 (6.2)
CarS_Pa			<1	<1					2.1 (1.2)					<1	**15.9** (3.6)			**19.7**	65.9 (21.7)	26.6 (8.6)
GerS_Ob	14.3 (13.8)																	**14.3**	25.0 (24.4)	22.8 (12.3)
GerS_Ct	14.3 (8.4)																	**14.3**	6.5 (2.2)	20.9 (10.5)
RLinS_Aa				1.3 (1.6)														**1.3**	10.8 (8.4)	27.3 (12.7)

Table S6: Overview of all linear monoterpenoids produced. Highest titres observed and the mTC/S responsible are shown. Averages of at least 3 biological replicates and the corresponding standard deviations are shown.

**Linear monoterpenoids**			
**Target linear compounds**	**Structure**	**Titre (mg L_{org}^{-1})**	**Strain + mTC/S**
geraniol	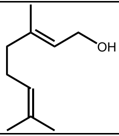	162.6 ± 98.2	GerS_Pc
(*E*)-β-ocimene	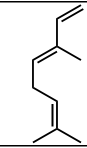	57.3 ± 56.9	OciS_Am
β-myrcene	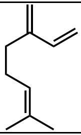	17.3 ± 15.5	CinS_At
linalool	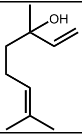	0.8 ± 1.4	CarS_Pa
**Additional linear compounds**			
nerol	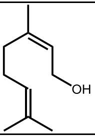	73.2 ± 61.8	GerS_Pc
geranial	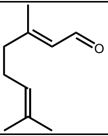	56.1 ± 35.0	GerS_Pc
citronellol	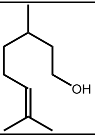	35.1 ± 19.7	GerS_Pc
neral	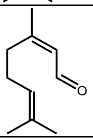	16.8 ± 7.2	GerS_Pc
citronellal	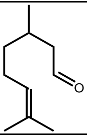	10.7 ± 2.0	GerS_Pc
iso-geraniol	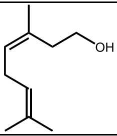	1.2 ± 1.4	GerS_Pc
(*Z*)-β-ocimene	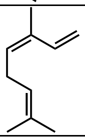	0.8 ± 1.7	OciS_Am

Table S7: Overview of all monocyclic monoterpenoids produced. Highest titres observed and the mTC/S responsible are shown. Averages of at least 3 biological replicates and the corresponding standard deviations are shown.

Monocyclic monoterpenoids			
**Target monocyclic compounds**	**Structure**	**Titre (mg L_{org}^{-1})**	**Strain + mTC/S**
(-)-(4*S*)-limonene*	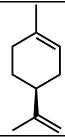	530.6 ± 102.3	SLimS_Ms
(+)-(4*R*)-limonene*	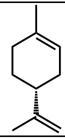	260.6 ± 122.2	RLimS_Cl
γ-terpinene	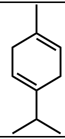	197.1 ± 119.7	gTerS_Ov
α-terpineol	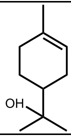	37.9 ± 36.3	aTerS_Mg
β-phellandrene	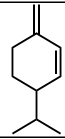	7.4 ± 3.5	(-)aPinS_Pt
terpinolene	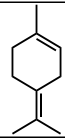	3.4 ± 0.5	CamS_Sl
**Additional monocyclic compounds**			
δ-terpineol	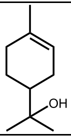	0.7 ± 1.3	CinS_At

* Stereoisomers identified based on published product profiles

Table S8: Overview of all bicyclic monoterpenoids produced. Highest titres observed and the mTC/S responsible are shown. Averages of at least 3 biological replicates and the corresponding standard deviations are shown.

Bicyclic monoterpenoids			
**Target bicyclic compounds**	**Structure**	**Titre (mg L_{org}^{-1})**	**Strain + mTC/S**
(-)-α-pinene*	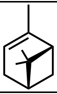	517.2 ± 132.7	(-)aPinS_Pt
(+)-α-pinene*	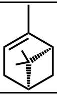	190.6 ± 31.9	(+)aPinS_Pt
(-)-β-pinene*	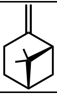	145.0 ± 33.5	(-)bPinS_Aa
1,8-cineole	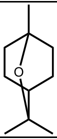	118.2 ± 153.6	CinS_Sf
sabinene	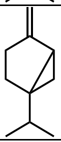	76.6 ± 74.0	SabS_Sp
fenchol	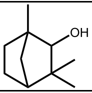	68.6 ± 30.9	FenS_Lv
3-carene	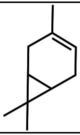	15.9 ± 3.6	CarS_Pa
camphene	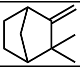	12.2 ± 3.4	CamS_Sl
**Additional bicyclic compounds**			
4-carene	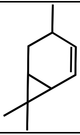	18.5 ± 12.7	gTerS_Ov
sabinene hydrate	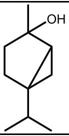	2.7 ± 4.1	CinS_Sf
thujene	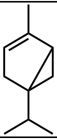	0.5 ± 0.1	CinS_Sf
borneol	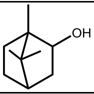	1.8 ± 0.1	CamS_Sl
pinan-2-ol	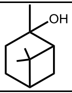	0.8 ± 0.7	(-)aPinS_Pt

* Stereoisomers identified based on published product profiles

Table S9: Overview of all additional terpenoids produced. Highest titres observed and the mTC/S responsible are shown. Averages of at least 3 biological replicates and the corresponding standard deviations are shown.

Sesquiterpenoids	Structure	Titre (mg L_{org}^{-1})	Strain + mTC/S
farnesol	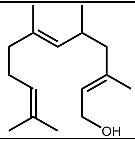	46.6 ± 38.9	PheS_Sl
farnesal	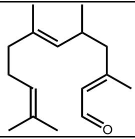	3.7 ± 2.1	SLimS_Ms
(*E*)-α-bisabolene	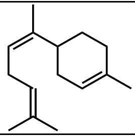	1.4 ± 2.4	CinS_At
farnesene	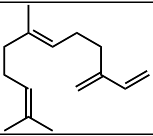	0.5 ± 0.9	CinS_At

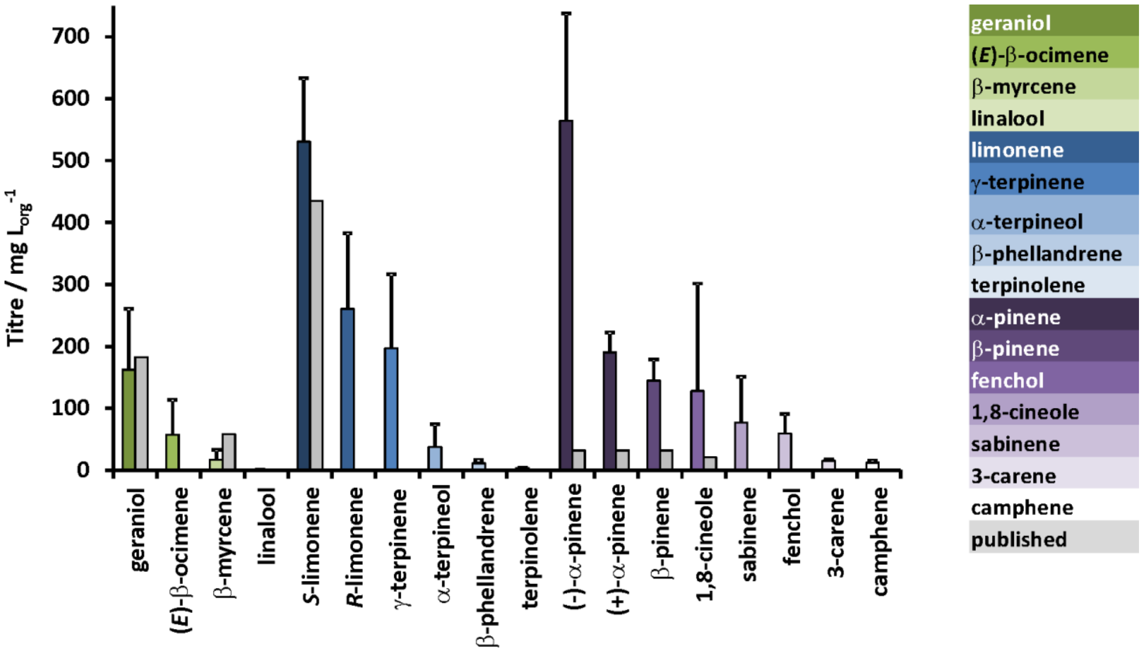

Figure S5: Titres produced in the platform vs. previously published titres. Monoterpenoid titres produced using our platform are shown in coloured bars, previously published titres are in grey bars. Published values: geraniol: 182.5 mg L^{-1} [39]; β-myrcene: 58.2 mg L^{-1} [40]; linalool: 0.1 mg L^{-1} [41]; limonene: 435 mg L^{-1} [1]; pinene: 32 mg L^{-1} [42]; 1,8-cineole: 21 mg L^{-1} [43]. Error bars represent the standard deviation of at least 3 biological replicates.

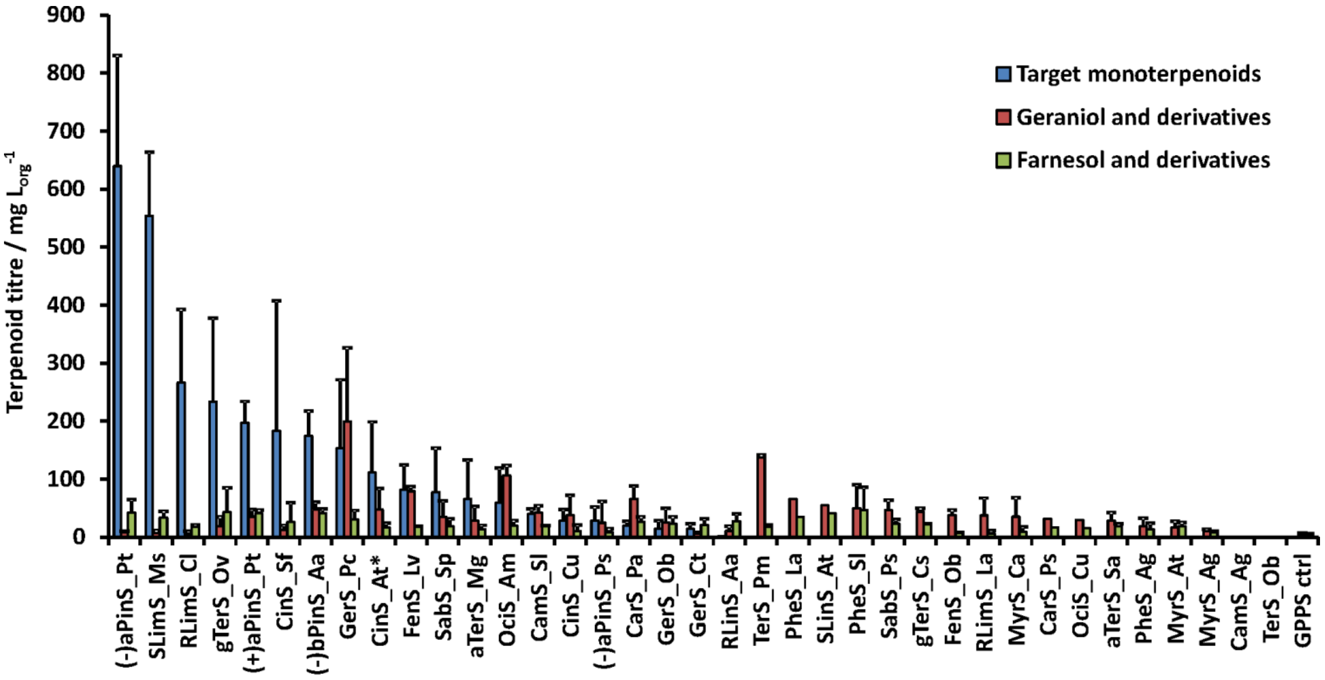

Figure S6: Monoterpenoid, geranoid and farnesol titres detected for each strain. Monoterpenoid titres produced using our platform are shown in blue bars, geraniol and derivatives in red bars, and farnesol and derivatives in green bars. Error bars represent the standard deviation of at least 3 biological replicates.

References

[1] J. Alonso-Gutierrez, R. Chan, T. S. Batth, P. D. Adams, J. D. Keasling, C. J. Petzold, T. S. Lee, *Metab Eng* **2013**, *19*, 33-41.

[2] T. S. Lee, R. A. Krupa, F. Zhang, M. Hajimorad, W. J. Holtz, N. Prasad, S. K. Lee, J. D. Keasling, *Journal of Biological Engineering* **2011**, *5*, 1-14.

[3] S. de Kok, et al., *ACS Synth Biol* **2014**, *3*, 97-106.

[4] D. C. Hyatt, B. Youn, Y. Zhao, B. Santhamma, R. M. Coates, R. B. Croteau, C. Kang, *Proc Natl Acad Sci U S A* **2007**, *104*, 5360-5365.

[5] a) A. Espah Borujeni, A. S. Channarasappa, H. M. Salis, *Nucleic Acids Res* **2014**, *42*, 2646-2659; b) H. M. Salis, E. A. Mirsky, C. A. Voigt, *Nat Biotechnol* **2009**, *27*, 946-950.

[6] F. R. Blattner, et al., *Science* **1997**, *277*, 1453-1462.

[7] K. Hayashi, et al., *Molecular Systems Biology* **2006**, *2*.

[8] M. Meselson, R. Yuan, *Nature* **1968**, *217*, 1110-1114.

[9] J. Bohlmann, M. Phillips, V. Ramachandiran, S. Katoh, R. Croteau, *Arch Biochem Biophys* **1999**, *368*, 232-243.

[10] V. Falara, et al., *Plant Physiol* **2011**, *157*, 770-789.

[11] J. Fäldt, D. Martin, B. Miller, S. Rawat, J. Bohlmann, *Plant Mol Biol* **2003**, *51*, 119-133.

[12] D. E. Hall, et al., *Plant J* **2011**, *65*, 936-948.

[13] S. C. Kampranis, et al., *Plant Cell* **2007**, *19*, 1994-2005.

[14] T. Shimada, T. Endo, H. Fujii, M. Hara, M. Omura, *Plant Science* **2005**, *168*, 987-995.

[15] F. Chen, D. K. Ro, J. Petri, J. Gershenzon, J. Bohlmann, E. Pichersky, D. Tholl, *Plant Physiol* **2004**, *135*, 1956-1966.

[16] Y. Iijima, R. Davidovich-Rikanati, E. Fridman, D. R. Gang, E. Bar, E. Lewinsohn, E. Pichersky, *Plant Physiol* **2004**, *136*, 3724-3736.

[17] M. Ito, G. Honda, *Phytochemistry* **2007**, *68*, 446-453.

[18] Y. Iijima, D. R. Gang, E. Fridman, E. Lewinsohn, E. Pichersky, *Plant Physiol* **2004**, *134*, 370-379.

[19] T. Yang, J. Li, H. X. Wang, Y. Zeng, *Phytochemistry* **2005**, *66*, 285-293.

[20] J. Lücker, M. K. El Tamer, W. Schwab, F. W. Verstappen, L. H. van der Plas, H. J. Bouwmeester, H. A. Verhoeven, *Eur J Biochem* **2002**, *269*, 3160-3171.

[21] C. Landmann, B. Fink, M. Festner, M. Dregus, K.-H. Engel, W. Schwab, *Archives of Biochemistry and Biophysics* **2007**, *465*, 417-429.

[22] S. M. Colby, W. R. Alonso, E. J. Katahira, D. J. McGarvey, R. Croteau, *J Biol Chem* **1993**, *268*, 23016-23024.

[23] J. W. Jia, J. Crock, S. Lu, R. Croteau, X. Y. Chen, *Arch Biochem Biophys* **1999**, *372*, 143-149.

[24] F. Chen, D. Tholl, J. C. D'Auria, A. Farooq, E. Pichersky, J. Gershenzon, *Plant Cell* **2003**, *15*, 481-494.

[25] J. Bohlmann, C. L. Steele, R. Croteau, *J Biol Chem* **1997**, *272*, 21784-21792.

[26] L. Del Terra, V. Lonzarich, E. Asquini, L. Navarini, G. Graziosi, F. Suggi Liverani, A. Pallavicini, *Phytochemistry* **2013**, *89*, 6-14.

[27] J. Bohlmann, D. Martin, N. J. Oldham, J. Gershenzon, *Arch Biochem Biophys* **2000**, *375*, 261-269.

[28] N. Dudareva, D. Martin, C. M. Kish, N. Kolosova, N. Gorenstein, J. Faldt, B. Miller, J. Bohlmann, *Plant Cell* **2003**, *15*, 1227-1241.

[29] Z. A. Demissie, L. S. Sarker, S. S. Mahmoud, *Planta* **2011**, *233*, 685-696.

[30] A. L. Schilmiller, I. Schauvinhold, M. Larson, R. Xu, A. L. Charbonneau, A. Schmidt, C. Wilkerson, R. L. Last, E. Pichersky, *Proc Natl Acad Sci U S A* **2009**, *106*, 10865-10870.

[31] S. A. McKay, W. L. Hunter, K. A. Godard, S. X. Wang, D. M. Martin, J. Bohlmann, A. L. Plant, *Plant Physiol* **2003**, *133*, 368-378.

[32] M. A. Phillips, M. R. Wildung, D. C. Williams, D. C. Hyatt, R. Croteau, *Arch Biochem Biophys* **2003**, *411*, 267-276.

[33] S. Lu, R. Xu, J. W. Jia, J. Pang, S. P. Matsuda, X. Y. Chen, *Plant Physiol* **2002**, *130*, 477-486.

[34] M. Galata, L. S. Sarker, S. S. Mahmoud, *Phytochemistry* **2014**, *102*, 64-73.

[35] C. Crocoll, J. Asbach, J. Novak, J. Gershenzon, J. Degenhardt, *Plant Mol Biol* **2010**, *73*, 587-603.

[36] S. Lee, J. Chappell, *Plant Physiol* **2008**, *147*, 1017-1033.

[37] C. G. Jones, C. I. Keeling, E. L. Ghisalberti, E. L. Barbour, J. A. Plummer, J. Bohlmann, *Arch Biochem Biophys* **2008**, *477*, 121-130.

[38] D. P. Huber, R. N. Philippe, K. A. Godard, R. N. Sturrock, J. Bohlmann, *Phytochemistry* **2005**, *66*, 1427-1439.

[39] J. Zhou, C. Wang, S.-H. Yoon, H.-J. Jang, E.-S. Choi, S.-W. Kim, *Journal of Biotechnology* **2014**, *169*, 42-50.

[40] E. M. Kim, J. H. Eom, Y. Um, Y. Kim, H. M. Woo, *J Agric Food Chem* **2015**, *63*, 4606-4612.

[41] a) P. Amiri, A. Shahpiri, M. A. Asadollahi, F. Momenbeik, S. Partow, *Biotechnol Lett* **2015**, *38*, 503-508; b) Y. Deng, M. Sun, S. Xu, J. Zhou, *J Appl Microbiol* **2016**, *Accepted Article*, doi: 10.1111/jam.13105; c) J. Rico, E. Pardo, M. Orejas, *Appl Environ Microbiol* **2010**, *76*, 6449-6454.

[42] S. Sarria, B. Wong, H. Garcia Martin, J. D. Keasling, P. Peralta-Yahya, *ACS Synth Biol* **2014**, *3*, 466-475.

[43] J. J. Shaw, T. Berbasova, T. Sasaki, K. Jefferson-George, D. J. Spakowicz, B. F. Dunican, C. E. Portero, A. Narvaez-Trujillo, S. A. Strobel, *J Biol Chem* **2015**, *290*, 8511-8526.
